# Regulation of ERK basal and pulsatile activity control proliferation and exit from the stem cell compartment in mammalian epidermis

**DOI:** 10.1073/pnas.2006965117

**Published:** 2020-07-10

**Authors:** Toru Hiratsuka, Ignacio Bordeu, Gunnar Pruessner, Fiona M. Watt

**Affiliations:** ^a^Centre for Stem Cells and Regenerative Medicine, King’s College London, SE1 9RT London, United Kingdom;; ^b^Department of Mathematics, Imperial College London, SW7 2BZ London, United Kingdom;; ^c^Department of Applied Mathematics and Theoretical Physics, Centre for Mathematical Sciences, University of Cambridge, CB3 0WA Cambridge, United Kingdom;; ^d^The Wellcome Trust/Cancer Research UK Gurdon Institute, University of Cambridge, CB2 1QN Cambridge, United Kingdom

**Keywords:** ERK, stem cells, keratinocytes, live imaging, cell signaling

## Abstract

Fluctuation in signal transduction pathways is frequently observed during mammalian development. However, its role in regulating stem cells has not been explored. Here we tracked spatiotemporal ERK MAPK dynamics in human epidermal stem cells. While stem cells and differentiated cells were distinguished by high and low stable basal ERK activity, respectively, we also found cells with pulsatile ERK activity. Transitions from Basal^hi^-Pulse^lo^ (stem) to Basal^hi^-Pulse^hi^, Basal^mid^-Pulse^hi^, and Basal^lo^-Pulse^lo^ (differentiated) cells occurred in expanding keratinocyte colonies and in response to differentiation stimuli. Pharmacological inhibition of ERK induced differentiation only when cells were in the Basal^mid^-Pulse^hi^ state. Basal ERK activity and pulses were differentially regulated by DUSP10 and DUSP6, leading us to speculate that DUSP6-mediated ERK pulse down-regulation promotes initiation of differentiation, whereas DUSP10-mediated down-regulation of mean ERK activity promotes and stabilizes postcommitment differentiation. Levels of MAPK1/MAPK3 transcripts correlated with DUSP6 and DUSP10 transcripts in individual cells, suggesting that ERK activity is negatively regulated by transcriptional and posttranslational mechanisms. When cells were cultured on a topography that mimics the epidermal−dermal interface, spatial segregation of mean ERK activity and pulses was observed. In vivo imaging of mouse epidermis revealed a patterned distribution of basal cells with pulsatile ERK activity, and down-regulation was linked to the onset of differentiation. Our findings demonstrate that ERK MAPK signal fluctuations link kinase activity to stem cell dynamics.

## Significance

Understanding how intracellular signaling cascades control cell fate is a key issue in stem cell biology. Here we show that exit from the stem cell compartment in mammalian epidermis is characterized by pulsatile ERK MAPK activity. Basal activity and pulses are differentially regulated by DUSP10 and DUSP6, two phosphatases that have been shown previously to regulate differentiation commitment in the epidermis. ERK activity is controlled both transcriptionally and posttranscriptionally. Spatial segregation of mean ERK activity and pulses is observed both in reconstituted human epidermis and in mouse epidermis. Our findings demonstrate the tight spatial and temporal regulation of ERK MAPK expression and activity in mammalian epidermis.

Fluctuation in signals involving Notch, Wnt, FGF, p53, NF-κB, and other pathways plays a significant role in mammalian development and physiology (1–6). Spatial and temporal heterogeneity of the signaling profiles in individual cells regulates gene expression and cell fate. This heterogeneity reflects changes in the intracellular and extracellular environment, including biochemical noise in signaling components, the availability and gradient of growth factors, and interactions with surrounding cells.

Spatiotemporal activation patterns of extracellular signal-regulated kinase (ERK) MAPK are believed to play a significant role in a variety of cellular processes, including cell proliferation, migration, and differentiation (7, 8). Recent single-cell live imaging approaches have revealed fluctuating and propagating features of ERK activation (9–13). In the epidermis of living mice, bursts of ERK activity radially propagate to neighboring cells and can be triggered by wounding and other external stimuli (11).

Stem cells reside in the basal layer of the epidermis, where they self-renew or generate committed cells that undergo terminal differentiation. Stem cell markers include high levels of α6 and β1 integrins (14, 15), Delta-like 1 (DLL1), and Lrig1 (16, 17). Despite the requirement of ERK activity to maintain epidermal stem cells (18–22), its role in cell state transitions, such as proliferative/quiescent and differentiation commitment, has not been studied.

Here we show, by live imaging of thousands of human epidermal cells, that there are dynamic transitions in ERK activity during stem cell colony expansion and differentiation. ERK pulse activity and basal levels are independently regulated by DUSP6 and DUSP10, components of the autoregulatory protein phosphatase network that acts as a switch between the stem cell state and the differentiated cell state (23). We also observe spatial segregation of cells with different ERK activity patterns on substrates mimicking the human epidermal−dermal interface and in live mouse skin, establishing the physiological significance of our observations.

## Results

### Transitions in ERK Activity Dynamics during Expansion of Stem Cell Colonies.

**Fig. 1.**
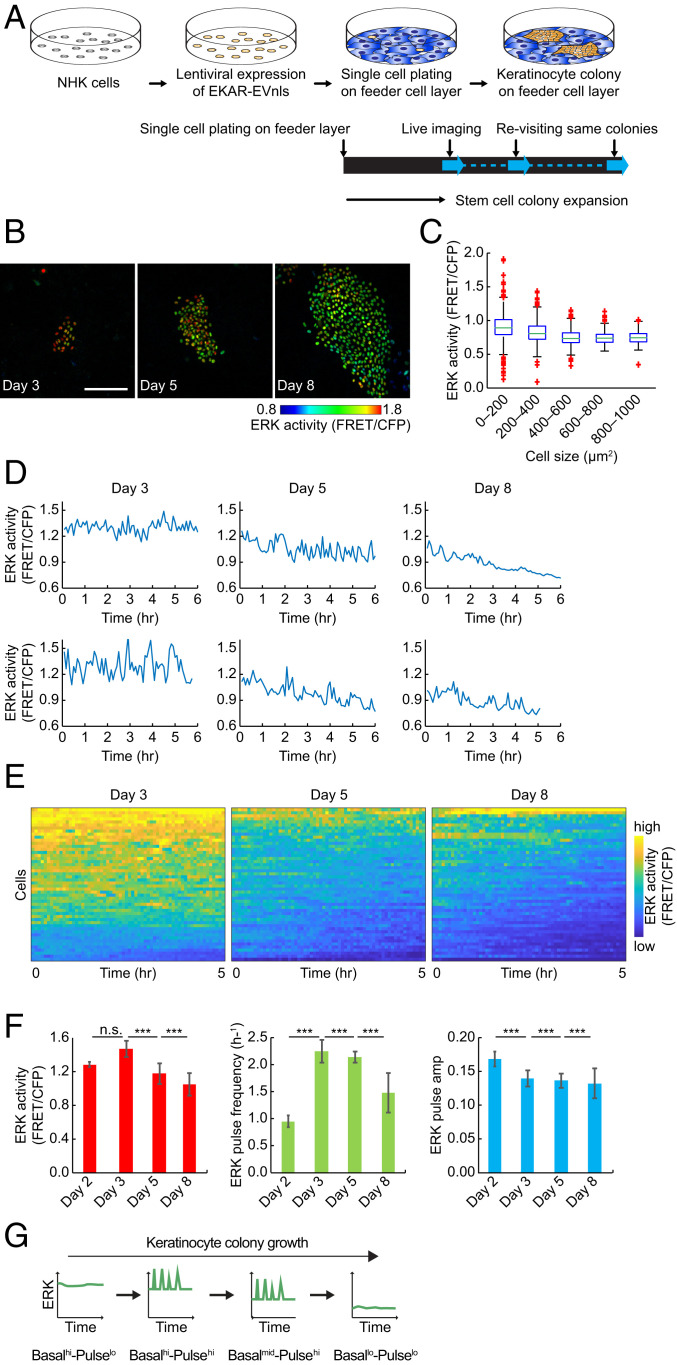
ERK activity dynamics transition in human epidermal stem cells. (*A*) Schematic of experimental setting. Primary NHKs were lentivirally transfected with EKAR-EVnls ERK FRET sensor and allowed to form colonies on feeder cell layers. The same NHK colonies were repeatedly observed for live imaging. (*B*) Representative Images of NHKs expressing EKAR-EVnls. The same colonies were observed at different days. Colors indicate ERK activity. (Scale bar: 200 μm.) (*C*) Box plots of single-cell ERK activity grouped by cell size: midline, median; box, 25th to 75th percentiles; whiskers, lower and higher extremes; red crosses, outliers (*n* = 3,581 cells). (*D*) Representative time series of ERK activity on different days. (*E*) Heat map of ERK activity over time for 50 cells ordered by descending mean ERK activity (FRET/CFP) over time. Colors indicate ERK activity. (*F*) Basal ERK activity (*Left*), ERK pulse frequency (*Middle*), and ERK pulse amplitude (*Right*) on different days. Data are shown as mean ± SEM (*n* = 542 cells for day 2, 3,323 cells for day 3, 11,527 cells for day 5, and 37,320 cells for day 8 cells, two-tailed unpaired Student’s *t* test; *P* values are indicated by ***P < 0.001; n.s., not significant (*P* > 0.05). (*G*) Schematic representation of transitions in ERK activity dynamics comprising basal ERK activity and pulses during stem cell colony growth.

We live imaged ERK activity in individual primary human neonatal keratinocytes (HNKs) via lentiviral expression of a nuclear-tagged fluorescence resonance energy transfer (FRET) biosensor for ERK, EKAR-EVnls (24). NHKs were seeded on a 3T3 feeder layer (25), and expanding colonies were subjected to live imaging on different days from 3 d to 8 d after plating (Fig. 1 *A*–*C*). Within colonies, mean ERK activity (hereinafter referred to as “basal activity”) was lower in large (median in Fig. 1*C*) than in small keratinocytes. Keratinocytes are known to enlarge as they undergo differentiation, and the proportion of differentiated cells increases as colonies expand. Therefore this observation is consistent with the previously reported down-regulation of basal ERK activity in differentiated cells (14). We also observed that there was a wider range of ERK activity in smaller than in large cells (whiskers in Fig. 1*C*).

In order to examine ERK activity dynamics in detail, we observed thousands of cells in growing keratinocyte colonies (Fig. 1 *D* and *E* and Movie S1; *n* = 3,323 cells for day 3, 11,527 cells for day 5, 37,320 cells for day 8). We found that ERK dynamics were characterized by pulses of activation as well as changes in basal activity. We measured the quantitative features of ERK activity pulses as local peaks (*SI Appendix*, Fig. S1*A*). We ruled out the possibility that the pulses were an imaging artifact by using a negative control FRET biosensor EKAREV-TA-nls, which has a mutation that results in loss of recognition by active ERK (*SI Appendix*, Fig. S1*B*) (26). NHKs expressing EKAREV-TA-nls showed almost no pulses: only 5% of cells showed very rare (0.003 pulses per hour) pulses (*SI Appendix*, Fig. S1*B*). The average duration of pulse activation in EKAR-EVnls−expressing cells was 0.25 h, which is consistent with that previously reported in immortalized epithelial cells (9, 10) (*SI Appendix*, Fig. S1*C*). The average pulse-to-pulse interval was 1.52 h. Notably, the histogram of interpulse intervals followed an exponential decay curve, which indicates that ERK pulses are stochastic rather than precisely timed events such as oscillations (*SI Appendix*, Fig. S1*D*).

The frequency of pulses in NHKs ranged from zero to ∼4.5 pulses per hour (Fig. 1*E* and *SI Appendix*, Fig. S1*E*). The ERK pulse frequency was high (>2.0 pulses per hour) on day 3 and day 5 of colony growth and subsequently reduced on day 8, while basal ERK activity gradually decreased (Fig. 1*F* and *SI Appendix*, Fig. S1 *E* and *F*). In contrast, although ERK pulse amplitude decreased over the period, the absolute reduction was very small (<0.005 FRET/ cyan fluorescent protein [CFP]; Fig. 1*F*), in line with previous studies in immortal cell lines (9, 10).

Detailed histogram analysis of basal ERK activity showed two peaks on day 3 (*SI Appendix*, Fig. S1*F*, asterisks). The ERK activity in the lower peak matched that of the peak on day 5 (*SI Appendix*, Fig. S1*F*). This suggested that the cell population with higher basal ERK activity on day 3 transited to the one with lower activity on day 5 rather than there being a gradual reduction in ERK activity within the whole cell population.

We extended our analysis to include day 2 colonies (*n* = 542 cells). NHKs on day 2 showed significantly lower ERK pulse frequency and higher basal activity compared to older colonies (Fig. 1*F* and *SI Appendix*, Fig. S1 *G*–*J*). We performed correlation analysis to determine whether basal ERK activity might influence ERK pulse activations. This revealed a moderate correlation on days 2, 3, and 8 (*R* = 0.23 to 0.65) but not on day 5 (*R* = 0.018) (*SI Appendix*, Fig. S1*K*), strengthening the conclusion that ERK activity dynamics on day 5 are distinct from those on day 3.

These analyses show that individual human epidermal cells not only differ in basal ERK activity but also exhibit pulses of activation. Based on the analysis of individual cells in colonies at different times after plating, we propose that stem cells have a Basal^hi^-Pulse^lo^ ERK profile, then transit to Basal^hi^-Pulse^hi^, to Basal^mid^-Pulse^hi^, and, finally, to Basal^lo^-Pulse^lo^ once they have undergone differentiation (Fig. 1*G*).

### Pulsatile ERK Activation Is Associated with Proliferation, whereas Pulse Down-Regulation Precedes Differentiation.

**Fig. 2.**
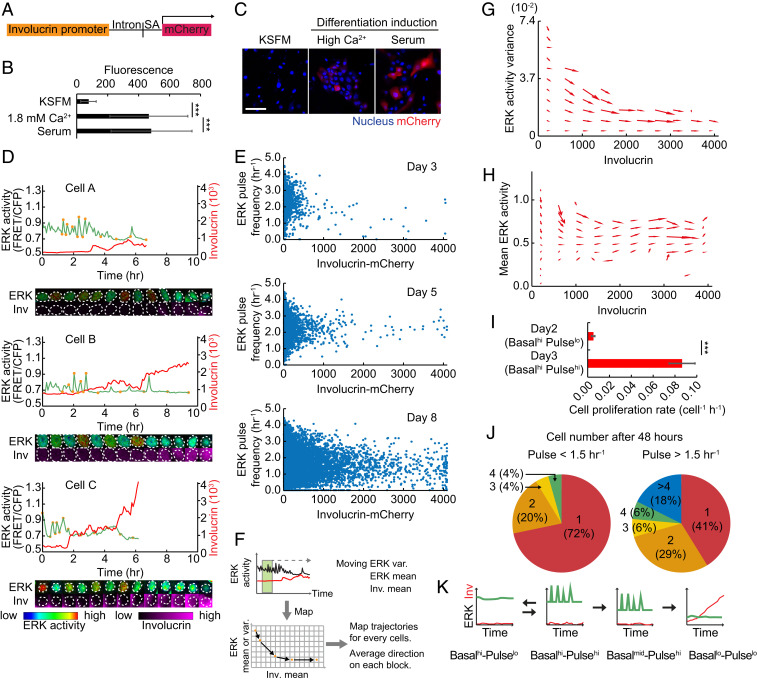
ERK pulse down-regulation precedes commitment to differentiation. (*A*) Construction of Involucrin-mCherry reporter. SA, SV40 splice acceptor. (*B* and *C*) Involucrin-mCherry reporter expression in cells cultured under the indicated conditions. Data are mean ± SD (*n* = 50 cells each). (Scale bar: 100 μm.) (*D*) Representative time series of ERK activity (green) and Involucrin-mCherry expression (red), and images of the cells at the time points indicated by the orange circles in each time series. Images are shown by the indicated lookup tables below. (*E*) Dot plots of ERK pulse frequency and Involucrin-mCherry expression in HNK cells on different days (*n* = 3,323 cells for day 3, 11,527 cells for day 5, and 37,320 cells for day 8 cells). (*F*) Schematics of the methodology for constructing the phase diagram of ERK activity. ERK moving variance and ERK and Involucrin moving mean levels are measured for each cell. Mapping of each ERK measure in relation to Involucrin generates a trajectory of the coevolution of the two factors. (*G* and *H*) Phase diagram of ERK (*G*) activity variance and (*H*) mean activity against mean Involucrin expression obtained from *n* = 3,397 cells. Arrows indicate the direction of transition for each compartment. (*I*) The proliferation rate of NHK cells in the ERK Basal^hi^-Pulse^lo^ state (day 2) and Basal^hi^-Pulse^hi^ state (day 3). Cell divisions were counted in time series images, and the increase in cell number was divided by the imaging period. Data are mean ± SD (*n* = 542 cells for day 2 and 3,323 cells for day 3). (*J*) Cell proliferation assay of single cells with ERK pulses lower (*Left*) or higher (*Right*) than 1.5 pulses per hour. Single cells were initially imaged to measure their pulse levels, and then the same cells were observed after 48 h. The number of daughter cells at 48 h and their respective fractions are indicated. (*K*) Schematic representation of modulation in ERK activation pulses during differentiation. Statistical significance was examined by two-tailed unpaired Student’s *t* test; *P* values are indicated by ***P < 0.001.

To confirm that transitions in ERK activity correlated with differentiation, we generated a fluorescent reporter of Involucrin, a marker gene that is up-regulated in differentiating suprabasal epidermal cells. We used the previously characterized Involucrin promoter and intron sequence (27) to drive mCherry expression (Fig. 2*A*). Increasing the extracellular Ca^2+^ concentration from 0.1 mM to 1.6 mM or serum stimulation can be used to stimulate keratinocyte stratification and differentiation; however, our culture system (Fig. 1*A*) contains high Ca^2+^ and serum. Therefore, we cultured keratinocytes in low Ca^2+^ serum-free medium (KSFM) to validate the Involucrin reporter (28). Human epidermal stem cells lentivirally expressing the Involucrin-mCherry reporter were induced to differentiate by changing the medium from KSFM (28) to medium containing high Ca^2+^ or serum. NHKs expressing the Involucrin-mCherry reporter showed significant mCherry induction following both differentiation stimuli (Fig. 2 *B* and *C*).

ERK activity and differentiation were simultaneously monitored by coexpression of the EKAR-EVnls and Involucrin reporters in individual keratinocytes. We found that ERK pulses were down-regulated coincident with the onset of Involucrin expression (Fig. 2*D*), while cells that maintained low or high Involucrin expression showed stable ERK activity profiles (*SI Appendix*, Fig. S2 *A* and *B*). As expected, during NHK colony growth, the number of cells expressing Involucrin-mCherry increased, and they tended to have lower ERK basal activity and pulse frequencies (Fig. 2*E* and *SI Appendix*, Fig. S2*C*). The reduction in pulse frequency, however, did not show a strong correlation with Involucrin-mCherry expression levels in individual cells. (Fig. 2*E*).

To further dissect the change in ERK activity associated with differentiation, we analyzed the trajectories of ERK activity (mean and variance) and Involucrin expression over time (Fig. 2*F*). We observed coevolution of ERK activity variance and Involucrin expression, with a strong tendency for undifferentiated cells to down-regulate pulse frequencies coupled with differentiation (Fig. 2*G*). In contrast, there was a gradual convergence of basal ERK activity toward a low level as differentiation proceeded (Fig. 2*H*). This difference suggests that ERK pulses and mean levels are subject to distinct regulatory mechanisms, and that the down-regulation in ERK pulses has a role in switching stem cells to differentiated cells. The trajectory of ERK dynamics in Basal^hi^-Pulse^hi^ (day 3 after plating) cells showed that the cells tend to decrease pulse frequency and retain mean activity, leading to Basal^hi^-Pulse^lo^ ERK activity (*SI Appendix*, Fig. S2*D*). This suggests that ERK dynamics are partially reversible in the early stage and irreversible once cells are committed to differentiation.

Epidermal keratinocytes not only transition from the stem cell compartment to the differentiation compartment but can also transition between cell division and quiescence (14). To determine whether ERK pulse activations were associated with cell division, we followed the fate of Basal^hi^-Pulse^lo^ (day 2 after plating) and Basal^hi^-Pulse^hi^ (day 3 after plating) cells that did not express Involucrin (Fig. 2*E*) by time-lapse imaging. Basal^hi^-Pulse^hi^ cells had a high probability of dividing, whereas Basal^hi^-Pulse^lo^ cells did not (Fig. 2*I*).

We also recorded the ERK pulse frequency of individual cells and whether or not they subsequently divided (within 48 h). We found that 70% of cells with low ERK pulse frequency (<1.5 pulses per hour) remained as single cells (*SI Appendix* and Fig. 2 *J*, *Left*), whereas 60% of cells with high ERK pulse frequency (>1.5 pulses per hour) proliferated, giving rise to two or more cells during the recording period (Fig. 2 *J*, *Right*). This indicates that cells with a pulsatile ERK profile are more likely to divide than cells with a stable high ERK profile.

These results, combined with our observations of cells expressing Involucrin-mCherry, suggest that stem cells transition from Basal^hi^-Pulse^lo^ to Basal^hi^-Pulse^hi^ during a proliferative phase, and that a subsequent transition to Basal^mid^-Pulse^hi^ is associated with differentiation, leading to Basal^lo^-Pulse^lo^ ERK activity in differentiated cells (Fig. 2*K*).

### ERK Pulse Modulation by Terminal Differentiation Stimuli.

**Fig. 3.**
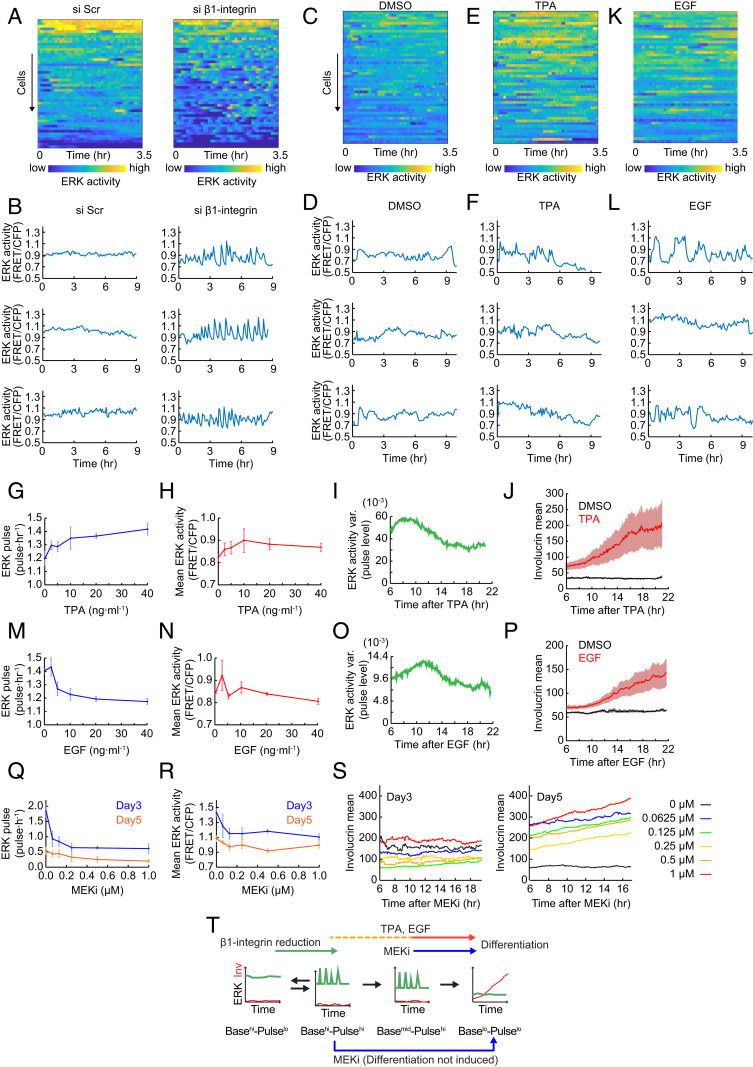
ERK pulse dynamics during cell differentiation. (*A*, *C*, *E*, and *K*) Heat maps of ERK activity over time for 50 cells ordered by descending overall mean ERK activity in each culture condition. Colors indicate ERK activity. (*B*, *D*, *F*, and *L*) Representative time series of ERK activity in cells treated with the indicated reagents. (*G* and *H*) Dose dependency of ERK (*G*) pulse frequency and (*H*) mean activity on TPA treatment. Data are shown as mean ± SD (*n* = 1,071 ± 27 cells for each condition). (*I* and *O*) ERK activity variance over time (see *Materials and Methods*) in cells treated with (*I*) 10 ng/mL TPA or (*O*) 10 ng/mL EGF. (*J* and *P*) Involucrin expression over time in cells treated with DMSO and (*J*) 10 ng/mL TPA or (*P*) 10 ng/mL EGF. Data are shown as mean ± SD. (*M* and *N*) Dose dependency of ERK (*M*) pulse frequency and (*N*) mean activity on EGF treatment. Data are shown as mean ± SD (*n* = 937 ± 158 cells for each condition). (*Q* and *R*) Dose dependency of ERK (*Q*) pulse frequency and (*R*) mean activity on MEK inhibitor PD0325901 treatment in NHKs cultured for 3 d or 5 d. Data are shown as mean ± SD (*n* = 670 ± 149 cells for each condition on day 3 and 885 ± 438 cells for each condition on day 5). (*S*) Involucrin expression over time in cells treated with the indicated dose of MEK inhibitor, PD0325901. Data are shown as mean ± SD (*n* = 670 ± 149 cells for each condition on day 3 and 885 ± 438 cells for each condition on day 5). (*T*) Schematic representation of the effects of differentiation stimuli an ERK activity dynamics.

We next tested the effect of different differentiation stimuli on ERK activity dynamics. Integrin-mediated adhesion maintains the stem cell state via ERK signaling (29). We found that β1 integrin small interfering RNA (siRNA) strongly induced ERK pulses in the whole cell population compared to control scrambled siRNA (Fig. 3 *A* and *B* and *SI Appendix*, Fig. S3 *A*–*C*). In contrast, blocking cell−cell adherens junctions and desmosomes by Ca^2+^ depletion of FAD medium (see *Materials and Methods* for composition) had little effect on ERK pulses (*SI Appendix*, Fig. S3 *D*–*H*, 1.15 pulses per hour vs. 1.06 pulses per hour). Consistent with this, Involucrin expression was not affected by the inhibition of intercellular adhesion (*SI Appendix*, Fig. S3*I*) (30). These results indicate that modulation of cell−substrate interaction plays a more significant role than Ca^2+^-mediated cell−cell interaction, and provides further experimental evidence of the transition in temporal ERK patterns during differentiation (Fig. 1*F*).

Although Ca^2+^-mediated adhesion did not affect ERK pulses, NHKs cultured at high density (2 × 10^5^/cm^2^) in KSFM showed higher ERK pulse frequency than those at low density (2 × 10^4^/cm^2^) (*SI Appendix*, Fig. S3 *J*–*L*). This suggests that, in addition to integrin-mediated adhesion, paracrine signaling may activate ERK pulses.

TPA (12-*O*-tetradecanoylphorbol-13-acetate) is known to stimulate Involucrin expression (31) and also increases ERK activity (32). When cells were stimulated with 10 ng/mL TPA, they exhibited highly pulsatile ERK activity (Fig. 3 *C*–*F*). Overall ERK pulse frequency increased in a dose-dependent manner up to 40 ng/mL TPA (Fig. 3*G*), while mean ERK levels peaked at 10 ng/mL (Fig. 3*H*). The time course analysis revealed that TPA induction of ERK pulses was transient, peaking at 9 h after the start of treatment (Fig. 3*I*). As before (Fig. 2 *G* and *K*), the onset of Involucrin expression coincided with the down-regulation of ERK pulses (Fig. 3*J*).

Like TPA, epidermal growth factor (EGF) transiently enhanced ERK pulse levels (Fig. 3 *K* and *L*), although, in contrast to TPA, EGF decreased overall ERK pulse frequency in a dose-dependent manner without affecting ERK mean levels (Fig. 3 *L* and *M*). ERK pulses peaked at 11 h after EGF treatment and then showed a significant decrease, again coincident with the onset of Involucrin expression (Fig. 3 *O* and *P*). Thus TPA and EGF had different effects on overall ERK pulse frequency (Fig. 3 *G* and *M*), but the time course of changes was similar, and, in both cases, ERK was down-regulated when cells expressed Involucrin (Fig. 3 *I*, *J*, *O*, and *P*). This leads us to speculate that the down-regulation of ERK pulses triggers differentiation (Fig. 2*K*).

When human keratinocytes were treated with the MEK inhibitor PD0325901 (MEKi), they exhibited a dose-dependent reduction in both ERK pulse frequency and basal levels (Fig. 3 *Q* and *R* and *SI Appendix*, Fig. S4 *A*–*D*). We tested the inhibitor on different days after plating cells: day 3 (correlating with Basal^hi^-Pulse^hi^; Fig. 1*F* and *SI Appendix*, Fig. S1 *E* and *F*) and day 5 (correlating with Basal^mid^-Pulse^hi^, Fig. 1*F* and *SI Appendix*, Fig. S1 *E* and *F*) (*SI Appendix*, Fig. S4 *E* and *F*). Although, on both days, MEKi induced the Basal^lo^-Pulse^lo^ state, differentiation was only induced on day 5 (Fig. 3*S*). Control dimethyl sulfoxide (DMSO)-treated day 3 populations included 15.8% of Basal^hi^-Pulse^lo^ cells (*SI Appendix*, Fig. S4*G*), while 1 µM MEKi treatment induced differentiation in only 4.0% of cells (*SI Appendix*, Fig. S4*H*). This strongly suggests that MEKi does not induce differentiation in Basal^hi^-Pulse^lo^ cells. Rather, the MEKi results suggest that the order of transitions in ERK dynamics shown in Fig. 2*K* must be followed for differentiation to occur.

We conclude that three distinct differentiation stimuli—reduced integrin-mediated adhesion, TPA, and EGF—all trigger ERK pulses and subsequent ERK down-regulation, whereas inhibition of MEK reduces ERK basal levels directly. Furthermore, cells initiate differentiation by transiting through the Basal^mid^-Pulse^hi^ state (Figs. 1*F*, 2*K*, and 3T).

### Regulation of ERK Mean and Pulsatile Activity by Protein Phosphatases.

**Fig. 4.**
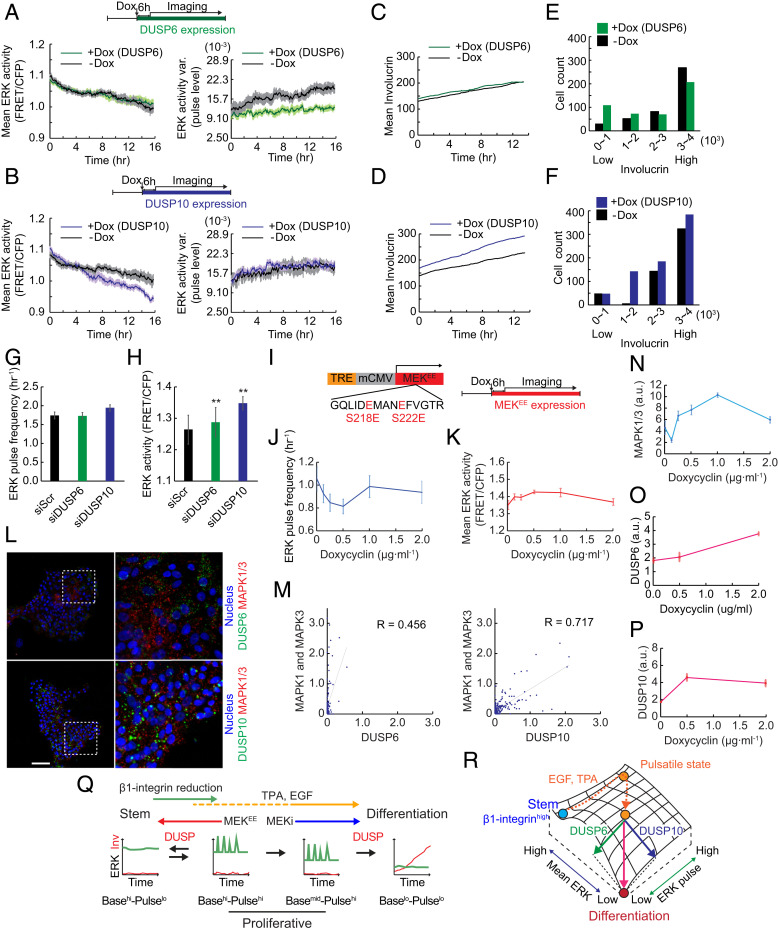
ERK pulse and mean levels are independently regulated by DUSP6 and DUSP10. (*A* and *B*) Mean (*Left*) and pulse levels (*Right*; see *Materials and Methods*) of ERK activity in NHKs treated with 1 µg/mL doxycyclin (green or purple) or vehicle (black). (*A*) DUSP 6 or (*B*) DUSP10 was induced by doxycycline treatment. Data are shown as mean ± SEM (*n* = 1,220 doxycyclin-treated cells and 1,261 vehicle-treated cells for *A*; *n* = 1,224 doxycyclin-treated cells and 1,005 vehicle-treated cells for *B*). (*C* and *D*) Involucrin expression over time in NHKs treated with 1 µg/mL doxycyclin (green or purple) or vehicle (black). (*C*) DUSP 6 or (*D*) DUSP10 was induced by the doxycycline treatment. (*E* and *F*) Cell numbers in the four bins of Involucrin-mCherry expression levels 18.5 h after doxycycline-induced DUSP6 and DUSP10 expression (green and purple, respectively) or vehicle (black) treatment. (*G* and *H*) ERK (*G*) pulse frequency and (*H*) mean ERK activity in NHKs treated with scrambled siRNA, siRNA against DUSP6, or siRNA against DUSP10. Data are shown as mean ± SEM (*n* = 5,701 siScr cells, 4,335 siDUSP6 cells, and 4,346 siDUSP10 cells, two-tailed unpaired Student’s *t* test; *P* values are indicated by **P < 0.01). (*I*) Construction of plasmid for doxycyclin-dependent expression of constitutively active MEK1 (MEK^EE^). For MEK^EE^ induction, cells were treated with 0.125 μg/mL to 2.0 µg/mL doxycyclin. (*J* and *K*) ERK (*J*) pulse frequency and (*K*) mean ERK activity of NHKs treated with doxycycline to induce MEK^EE^ expression. Data are shown as mean ± SEM (*n* = 1,140 ± 494 cells for each condition). (*L*) Representative images of RNA in situ hybridization of DUSP6 (*Upper*, green), DUSP10 (*Lower*, green), and MAPK1 and MAPK3 (red) transcripts. The *Right* images show enlarged views of the white-dotted squares in the *Left* images. (Scale bar: 100 μm.) (*M*) Dot plots and correlation analysis of DUSP6 (*Left*) or DUSP10 (*Right*), and MAPK1 and MAPK3 in situ hybridization signals in NHKs cultured for 5 d. Lines are regression lines, and values are Spearman’s rank correlation coefficient. Data are mean ± SEM (*n* = 66 cells for DUSP6 and 170 for DUSP10). (*N*–*P*) Quantification of RNA in situ hybridization signals of (*N*) MAPK1 and MAPK3, (*O*) DUSP6, and (*P*) DUSP10 in NHKs treated with doxycycline to induce MEK^EE^ expression. Data are mean ± SEM (*n* = 202 ± 82 cells for *N*, 219 ± 115 cells for *O*, and 174 ± 44 cells for *P*). (*Q*) Schematic representation of the regulation of ERK dynamics. (*R*) Schematic representation of the regulation of ERK mean and pulse levels by DUSP6 and DUSP10 during differentiation.

One likely mechanism by which ERK basal activity and pulses are controlled is via negative feedback regulation by protein phosphatases (33). We therefore examined the effects of DUSP6 and DUSP10, key members of the protein phosphatase network that acts as a commitment switch in human epidermal stem cells (23). By live imaging cells in which overexpression of each DUSP was induced by doxycycline, we found that DUSP6 reduced ERK pulses without changing basal ERK activity (Fig. 4*A*), while DUSP10 down-regulated basal ERK levels without changing ERK pulses (Fig. 4*B*). This indicates that mean ERK levels and ERK pulses are independently regulated by different phosphatases.

Whereas DUSP10 overexpression strongly stimulated Involucrin expression during the recording period, DUSP6 did not have a significant effect (Fig. 4 *C* and *D*). However, by binning cells into groups of low/intermediate/high Involucrin levels, we found that DUSP6 and DUSP10 induction had different effects on each population. DUSP6 increased the proportion of cells with low Involucrin expression (Fig. 4*E*). In contrast, DUSP10 increased the proportion of cells with high Involucrin expression (Fig. 4*F*). This suggests the intriguing possibility that DUSP6-mediated ERK pulse down-regulation promotes the initiation of differentiation, whereas DUSP10-mediated down-regulation of mean ERK activity promotes and stabilizes postcommitment differentiation.

We further analyzed the effect of DUSP6 on subpopulations binned by Involucrin-mCherry expression levels. In vehicle-treated control cells, the proportion of low Involucrin-mCherry cells decreased early (*SI Appendix*, Fig. S5*A*, black arrow), and middle-low and middle-high Involucrin-mCherry cells increased later (around 2 h and 6 h; *SI Appendix*, Fig. S5 *B* and *C*, black arrows), which led to an increase in the high Involucrin-mCherry cell population (*SI Appendix*, Fig. S5*D*). In sharp contrast, DUSP6 overexpression increased the low Involucrin-mCherry proportion early (around 2 h; *SI Appendix*, Fig. S5*A*, green arrow) but did not lead to the later increase of the proportion of cells with middle-low, middle-high, or high Involucrin-mCherry expression (*SI Appendix*, Fig. S5 *B*–*D*). This supports the conclusion that DUSP6 expression plays a role in the early phase of differentiation, consistent with the finding that DUSP6 is transiently up-regulated on commitment, while DUSP10 up-regulation is more sustained (23).

We also tested siRNA-mediated knockdown of DUSP6 and DUSP10 (23). Reduced DUSP6 expression did not have a significant effect on ERK pulse frequency or basal activity (Fig. 4 *G* and *H*). However, reduction in DUSP10 expression led to increased basal ERK activity while maintaining pulse frequency (Fig. 4 *G* and *H*). This confirms that DUSP10 controls basal ERK activity.

As a further means of perturbing ERK activity, we lentivirally transfected HNK cells with a constitutively active form of MEK1 (MEK^EE^). MEK1 lies immediately upstream of ERK in the ERK signaling cascade and can override the differentiation stimulus of reduced β1 integrin signaling in keratinocytes (34). Moderate induction of MEK^EE^ expression with 0.5 µg/mL doxycycline (Fig. 4*I*) significantly decreased ERK pulse frequency (Fig. 4*J*) and increased basal ERK activity (Fig. 4*K*), promoting the transition from Basal^hi^-Pulse^hi^ to Basal^hi^-Pulse^lo^ ERK.

### Transcriptional Control of ERK, DUSP6, and DUSP10.

Previous studies have demonstrated interactions between DUSP6, DUSP10, and ERK at the transcriptional level in addition to the posttranslational level (23, 35–37). We therefore compared transcripts of MAPK3 and MAPK1, which encode ERK1 and ERK2 proteins, together with DUSP6 and DUSP10, in individual cells using RNA fluorescence in situ hybridization (Fig. 4*L*). MAPK1/MAPK3 gene expression was significantly correlated with DUSP10 and DUSP6 gene expression (Fig. 4*M*). In addition, the level of MEK^EE^ induction that decreased ERK pulse frequency and increased basal ERK activity (Fig. 4 *J* and *K*) also increased expression of MAPK1, MAPK3, DUSP6, and DUSP10 transcripts (Fig. 4 *N*–*P*). We conclude that ERK activity in keratinocytes is subject to both transcriptional and posttranscriptional regulation and that there are compensatory mechanisms to prevent excessive upstream stimulation of ERK activity (Fig. 4*R*).

Together, β1 integrin, EGF and their downstream effectors trigger ERK dynamic transitions that achieve different cellular outcomes (Fig. 4*Q*). DUSP expression leads to Pulse^lo^ states whether basal ERK activity is high or low. The Base^hi^-Pulse^lo^ state could be the result of transcriptional up-regulation of MAPK1/MAPK3 that could potentially override the effect of DUSP10 in reducing basal ERK activity. During differentiation, DUSP6 and DUSP10 independently down-regulate ERK pulses and basal activity (Fig. 4*R*). The loss of correlation between ERK basal activity and pulse frequency in the Base^mid^-Pulse^hi^ state (*SI Appendix*, Fig. S1*K*) indicates independent down-regulation of ERK pulses and basal activity.

### Patterning of Keratinocytes with Different ERK Kinetics in Response to Substrate Topography.

**Fig. 5.**
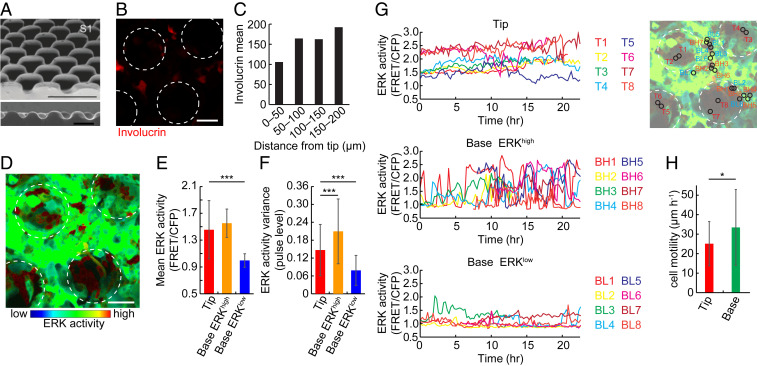
Spatial segregation of ERK activity patterns on topographical substrates. (*A*) Top (*Upper*) and cross-section (*Lower*) SEM images of the patterned PDMS substrate. Reproduced from a previous publication (39). (Scale bars: 400 μm [*Upper*] and 500 μm [*Lower*].) (*B*) Involucrin-mCherry expression on the patterned PDMS substrate. White dotted circles: tips of the substrate. (Scale bar: 100 μm.) (*C*) Mean Involucrin-mCherry expression in regions at different distances from the tips. (*D*) Representative image of ERK activity in human keratinocytes cultured on the PDMS substrate. Colors indicate ERK activity. White circles: tips of the substrate. (Scale bar: 100 μm.) (*E* and *F*) ERK (*E*) activity mean and (*F*) pulse levels of cells cultured on patterned substrates. Data are mean ± SD (*n* = 50 tip cells, 27 base ERK^high^ cells, and 23 base ERK^low^ cells, two-tailed unpaired Student’s *t* test; *P* values are indicated by ***P < 0.001). (*G*) (*Left*) Representative time series of human keratinocytes on tip regions (*Upper*) and base regions (troughs) with high (*Middle*) and low (*Lower*) ERK activity. Eight time series are shown for each group. The locations of the cells are mapped on the image to *Right*. (*H*) Motility of cells at the tip and base. Data are mean ± SD (*n* = 45 tip cells, 50 base ERK^high^ cells, two-tailed unpaired Student’s *t* test; *P* values are indicated by *P < 0.05).

We have previously reported that human epidermal stem cells have a patterned distribution in skin (38), leading us to predict that ERK dynamics would be spatially regulated. To examine this, we plated NHKs coexpressing a cytoplasmic ERK sensor (EKAR-EVnes) and Involucrin-mCherry on collagen-coated undulating polydimethylsiloxane (PDMS) substrates that mimic the topography of the human epidermal−dermal junction (23, 39). As in human epidermis, stem cells that express high β1 integrin and DUSP6 levels cluster at the tips of the features, whereas DUSP10 expression is more uniformly distributed (23). Once the cells had formed a confluent multilayered sheet, they were subjected to live-cell imaging (Fig. 5*A*). As reported previously (39), Involucrin-positive keratinocytes accumulated at the base of the features (troughs), while Involucrin-negative cells accumulated on the tips (Fig. 5 *B* and *C*).

We observed a patterned distribution of ERK activity on the substrates. Cells on the tips had higher basal ERK activity and lower ERK pulse frequencies than cells in the troughs (Fig. 5 *E*–*G* and Movie S2). Tip-located cells were also less motile (Fig. 5*H* and Movie S2), consistent with the high β1 integrin expression and low motility of epidermal stem cells (39). Conversely, cells in the troughs and sides of the substrates had low stable or pulsatile ERK activity (Fig. 5 *D*–*G* and Movie S2). Those cells in the troughs with high mean ERK activity had a higher level of ERK pulsatile activity than other cells (Fig. 5 *E*–*G*).

We conclude that on a three-dimensional (3D) topography that mimics the human epidermal−dermal interface cells with distinct patterns of ERK activity were differentially localized. The tip “stem cell niche” regions were occupied by cells with stable high ERK activity (40), while the base regions were occupied by cells with pulsatile ERK patterns or cells with low stable activity that underwent differentiation.

### ERK Pulse Kinetics Are Preserved in Mouse Epidermis.

**Fig. 6.**
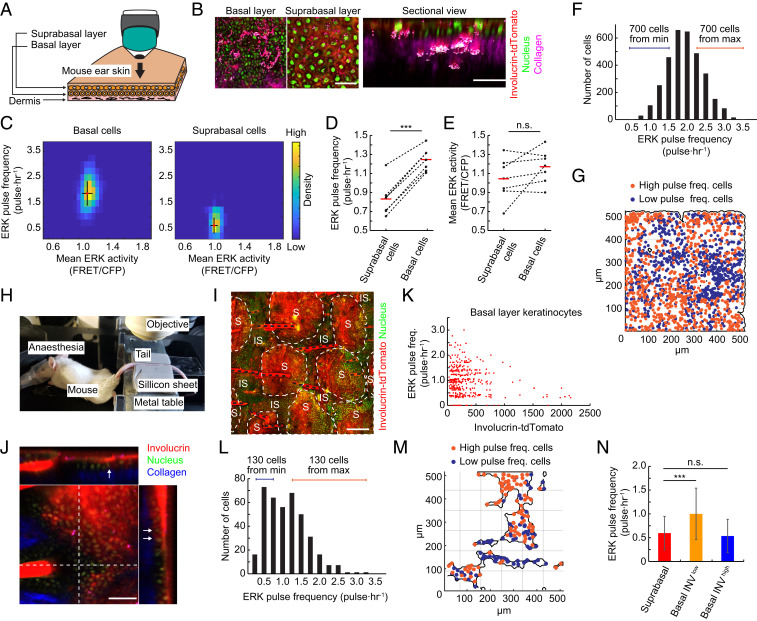
ERK pulse activity is observed in live mouse epidermis. (*A*) Schematic of in vivo observation of mouse epidermis. Ear skin of mouse epidermis expressing EKAR-EVnls and Involucrin-tdTomato was observed by multiphoton microscopy. (*B*) Representative images of epidermis in mouse expressing EKAR-EVnls and Involucrin-tdTomato. Collagen (magenta) was visualized by SHG microscopy. Sectional view image was reconstructed from *z*-stack images. (Scale bar: 100 μm.) (*C*) A 2D histogram of ERK pulse frequency and mean activity in basal (*Left*) and suprabasal (*Right*) layer cells. Mean and SD are shown by red dots and black lines, respectively (*n* = 3,238 basal cells and 352 suprabasal cells). (*D* and *E*) ERK (*D*) pulse frequency and (*E*) mean activity in the basal and suprabasal layer cells of seven mice. Mean is shown by red bars. (*F*) Distribution of ERK pulse frequencies of cells in the basal layer of mouse ear skin (*n* = 3,238 cells). (*G*) Mapping of basal layer keratinocytes showing the 700 least pulsatile (blue) and 700 most pulsatile (orange) cells, as indicated in *F*. (*H*) In vivo imaging of mouse tail epidermis. The anesthetized mouse was placed on a heated pad, and the tail was immobilized between soft silicon gum sheets. (*I*) Projection XY view of tail epidermis of mouse expressing EKAR-EVnls and Involucrin-tdTomato. S: scale epidermis; IS: interscale epidermis; black dotted lines: hair. (Scale bar: 200 μm.) (*J*) Orthogonal views of tail epidermis of mouse expressing EKAR-EVnls and Involucrin-tdTomato. Arrows indicate basal layer cells expressing Involucrin. (Scale bar: 100 μm.) (*K*) Dot plot of ERK pulse frequency and Involucrin-tdTomato expression in individual cells (*n* = 391 cells). (*L*) Distribution of ERK pulse frequencies of cells in the basal layer of mouse tail skin (*n* = 391 cells). (*M*) Mapping of basal layer keratinocytes showing the 130 least pulsatile (blue) and 130 most pulsatile (orange) cells as indicated in *L*. (*N*) Mean ERK pulse frequencies in the indicated tail epidermal cells. Data are mean ± SD (*n* = 318, 374, and 19 cells). Statistical significance was examined by two-tailed unpaired Student’s *t* test; *P* values are indicated by ***P < 0.001, n.s. = not significant (*P* > 0.05).

By live imaging of human epidermal cells, we found that down-regulation of pulsatile ERK activity preceded terminal differentiation and that ERK activity was patterned according to the location of stem cells and differentiated cells. To test whether this was also the case in living tissue, we generated mice that expressed both EKAR-EVnls and an Involucrin−tdTomato reporter (41). In interfollicular epidermis of mouse, as in human skin, the stem cells reside in the basal layer, and differentiating cells occupy the suprabasal layers (Fig. 6 *A* and *B*). We imaged two different regions of the skin, in which epidermal cells have distinct patterns of proliferation and differentiation: the ear (42) and tail (43).

In the skin of anesthetized mice, the boundary between the epidermis and the underlying dermis could readily be visualized by second harmonic generation (SHG) microscopy of collagen. Differentiating cells expressed tdTomato, and all cell nuclei expressed EKAR-EVnls (Fig. 6 *B*, *I*, and *J*). Time lapse observation of ERK activity revealed that basal keratinocytes had significantly higher ERK pulse levels than differentiating, tdTomato-positive suprabasal keratinocytes (Fig. 6*C* and *SI Appendix*, S6 *A* and *B*).

Quantitative analysis of ear epidermis revealed that there was a greater difference in ERK pulse levels (1.88 pulses per hour vs. 0.64 pulses per hour) than ERK mean levels (1.12 vs. 1.05 FRET/CFP) between basal and suprabasal cells (Fig. 6*C*). Observation of multiple mice showed consistent differences in ERK pulse levels between the basal and suprabasal layers, whereas the differences in ERK mean levels were relatively limited and highly variable among the seven mice examined (Fig. 6 *D* and *E*). This suggests a more significant role of ERK pulses than basal activity in the epidermis of living mice.

Ear epidermis is organized into columns of differentiated cells arranged above groups of basal cells, which have been referred to as epidermal proliferative units (EPUs) (42, 44). The width of an ear EPU is ∼25 μm diameter, with ∼8 to 10 cells in the basal layer. We noticed a high variance in ERK pulse frequencies in the basal layer of ear epidermis and therefore mapped the distribution of basal cells with high or low ERK pulse levels (Fig. 6*F*). This revealed that cells with low ERK pulse levels were clustered (Fig. 6*G* and *SI Appendix*, Fig. S6*C*). The cluster sizes were estimated to be about 50 μm (*SI Appendix*, Fig. S6*C*), which is similar to the reported EPU size (42, 44). This segregation of cell clusters with different ERK dynamics is reminiscent of spatial segregation of cells with different ERK profiles on substrates mimicking human epidermis (Fig. 5*D*).

In the interfollicular epidermis of mouse tail skin there are two distinct programs of terminal differentiation: scale (parakeratosis) and interscale (orthokeratosis) (43). The scale forms postnatally, and it has been speculated that postnatal expansion is limited by a subset of keratinocytes that express Involucrin in the basal layer of the interscale (45). This led us to predict that the spatial distribution of basal cells with pulsatile ERK activity would differ between tail and ear skin, and also enabled us to monitor Involucrin-positive cells in the basal layer of tail epidermis. We imaged the tails of mice expressing both EKAR-EVnls and the Involucrin-tdTomato reporter (Fig. 6 *H* and *I* and Movie S3) and confirmed that some basal layer keratinocytes expressed Involucrin (Fig. 6*J*, arrows). In vivo time-lapse imaging of the mice revealed that ERK pulses were significantly reduced in basal layer keratinocytes expressing Involucrin compared to Involucrin-negative basal cells (Fig. 6*K*).

In the tail epidermal basal layer, ERK Pulse^hi^-Involucrin^lo^ and ERK Pulse^lo^-Involucrin^hi^ cells were mostly intermingled (Fig. 6 *L* and *M*). In contrast to ear epidermis, there was no significant clustering of low or high ERK pulse cells (Fig. 6*M* and *SI Appendix*, Fig. S6*D*). We found that the average ERK pulse frequency in Involucrin^hi^ basal cells was comparable to that in suprabasal differentiated cells (Fig. 6*N*). This rules out the possibility that the reduced ERK pulses in Involucrin expressing cells of the ear are an artifact of imaging different layers of skin. It also confirms the strong coupling of reduced ERK pulses and Involucrin expression.

Together, our results indicate that ERK pulses are robustly coupled with cell fate, both in cultured human epidermal stem cells and in vivo mouse epidermal cells. The clustered localization and Basal^hi^-Pulse^lo^ stem cells in culture (Fig. 5*D*) and in mouse skin (Fig. 6*G* and *SI Appendix*, Fig. S6*C*) are fully compatible with the idea that stem cells reside in specific niches that modulate key signaling pathways (46).

## Discussion

We have demonstrated that basal and pulsatile ERK activation dynamically regulates epidermal stem cell fate. Previous studies have shown that ERK plays a key role in exit of embryonic stem cells from pluripotency and in lineage specification (47, 48), but the significance of cell-to-cell variation in ERK activity has been unclear. We show that ERK pulse frequencies are regulated independently of basal ERK activity, and that dynamic ERK activity is a feature of both cultured human epidermis and the epidermis of living mice.

ERK pulses have been reported in multiple cell types in relation to cell proliferation and tissue morphogenesis (9, 10, 13, 49, 50). Our research demonstrates that ERK pulses play significant roles in stem cell fate regulation and raises the intriguing possibility that pulse-mediated changes in cell fate are conserved in multiple tissues and organisms. Indeed, in vivo imaging approaches have shown pulsatile ERK activity in mouse mammary gland and intestine (10, 51, 52). It has previously been shown that Fra-1 expression in MCF-10A cells is controlled by integrative ERK activation rather than individual features such as the frequency, amplitude, or duration of pulsatile ERK activity (53). Whether this is also the case in the epidermis remains to be explored. It is also of interest to discover whether the relationship between ERK dynamics and Involucrin expression is conserved in the case of other keratinocyte differentiation markers, such as envoplakin, periplakin, and transglutaminase 1, which are up-regulated with different kinetics in the epidermis (54, 55).

Our finding of independent regulation of ERK pulse and mean levels by DUSP6 and DUSP10 indicates that DUSP6 and DUSP10 operate as distinct negative feedback regulators to achieve different ERK activity profiles and different cellular outcomes. Phosphatase family members are tightly regulated not only at the transcriptional level but also posttranscriptionally. When human keratinocytes commit to differentiation, a variety of phosphatases are induced and form an autoregulatory network (23). Furthermore, the half-life of DUSP6 messenger RNA and protein is very short (56) and controlled by the 3′ untranslated region (57). Therefore, the stoichiometry of different protein phosphatases may play a significant role in ERK dynamics and control of cell fate.

We also observed patterning of cells with different types of ERK activity on culture substrates that mimic the topography of the epidermal−dermal junction, consistent with the different patterns of DUSP6 and DUSP10 expression in the basal layer of human epidermis (23, 39). ERK activity was subject to both transcriptional and posttranscriptional regulation and subject to compensatory mechanisms to prevent excessive stimulation of ERK on constitutive activation of MEK1. In mouse epidermis, there was a clear difference in ERK pulse frequencies, but not basal levels, in the stem and differentiated cell layers. This suggests that ERK pulse modulation is a strategy for cells to switch states in vivo as well as in vitro. Differences in the spatial segregation of cells with different ERK profiles between ear and tail skin are likely to reflect differences in the architecture of the stem cell niche. Further studies are needed to map these to expression of specific phosphatases in mouse epidermis.

Fluctuations of signaling pathways are increasingly recognized as key determinants for tissue development (6). Multifaceted features of those fluctuations, such as phase, frequency, and amplitude, provide potentially different outputs in terms of cell fate. It will now be of significant interest to explore whether other signaling pathways show pulsatile activity in the epidermis and, if so, how they interact with ERK.

## Materials and Methods

### Reagents.

Hoechst 33342 was obtained from Molecular Probes, and TPA, EGF, and PD0325901 were obtained from Sigma. RNAscope probes against human MAPK1, MAPK3, DUSP6, and DUSP10 were purchased from Advanced Cells Diagnostics (Probe-Hs-MAPK1, catalog number 470741-C2; Probe-Hs-MAPK3, catalog number 470731-C2; Probe-Hs-DUSP6, catalog number 405361; and Probe-Hs-DUSP10, catalog number 583311).

### Constructs.

The pCSII-EKAREV-nls and pCSII-EKAREV-nes constructs were previously described (58). EKAREV-TA-nls, a negative control ERK FRET biosensor, was a kind gift from Eishu Hirata, Cancer Research Institute of Kanazawa, Japan, and was subcloned into pCSII vector. To construct pLenti-Involucrin-mCherry, the sequence comprising the full-length 3.7-kb Involucrin promoter, Involucrin intron, and SV40 splice donor and acceptor (S_D_S_A_) was subcloned from a previously reported beta-galactosidase reporter (59) into pLenti backbone, and mCherry was tagged to the carboxy (C) terminus of the promoter sequence. Doxycycline-inducible DUSP expression plasmids (pCW57-DUSP6 and pCW57-DUSP10) were previously described (23), except that GFP was removed from the vector for compatibility with EKAREV. The doxycycline-inducible MEK^EE^ expression plasmid (pCW57-MEK^EE^) was constructed by subcloning a constitutively active MEK1 mutant, MEK^EE^, into pCW57 vector (60).

### Mice.

Transgenic mice expressing EKAR-EVnls were obtained from the Laboratory Animal Resource Bank, National Institute of Biomedical Innovation, Health and Nutrition (61). Involucrin-tdTomato transgenic mice were obtained from the Institute of Molecular Genetics of the ASCR, v.v.i. All experimental procedures were carried out under the terms of a UK Home Office project license (PPL 70/8474) after local ethical review at King’s College London.

### Cell Culture.

Primary neonatal human keratinocytes (NHKs, strain km) were used in all experiments at passages 6 to 8. All cell stocks were routinely tested for mycoplasma contamination and were negative. The cells are not subjected to short tandem repeat (STR) profiling because they are not an established cell line. Cells were cultured on a mitotically inactivated feeder layer of J2-3T3 cells in FAD medium (one part Ham’s F-12, three parts Dulbecco’s modified Eagle’s medium [DMEM], 1.8 × 10^−4^ M adenine), supplemented with 10% fetal bovine serum (FBS) and 0.5 μg/mL hydrocortisone, 5 μg/mL insulin, 10^−10^ M cholera enterotoxin, and 10 ng/mL EGF (HICE mixture) (complete FAD medium) (17). For Ca^2+^-depleted keratinocyte culture medium, FBS was chelated with Chelex-100 resin (BioRad) and added to Ca^2+^-free FAD medium. J2-3T3 cells were cultured in high-glucose DMEM (Sigma-Aldrich) supplemented with 10% (vol/vol) adult bovine serum (Life Technologies). HEK293T cells were cultured in high-glucose DMEM (Sigma-Aldrich) supplemented with 10% FBS.

In some experiments, NHKs were plated on collagen-coated flasks (precoated with rat tail collagen type I [Corning], 20 µg/mL for 3 h) and cultured in feeder-free conditions in KSFM containing 30 µg/mL bovine pituitary extract (BPE) and 0.2 ng/mL EGF (Gibco). KSFM-cultured cells were stimulated to differentiate (Fig. 1 *F* and *G*) by exchanging the medium with high Ca^2+^ KSFM (1.2 mM) or complete FAD medium (containing 10% FCS).

Patterned PDMS substrates were precoated with rat tail collagen type I (Corning), 20 µg/mL for 3 h. Cells were seeded in complete FAD medium at a density of 75,000 cells per cm for 45 min at 37 °C. Substrates were rinsed gently once with FAD medium to remove nonadherent cells and transferred to 6-cm dishes containing inactivated J2-3T3 cells seeded at a density of 20,000 cells per cm^2^.

EGF, TPA, and PD0525901 were added to the medium 6 h prior to live imaging.

### Patterned PDMS Substrates.

Patterned PDMS substrates were generated as previously described with circle diameter (*d*) 150 µm, center-to-center distance (λ) 300 µm, and ultraviolet light exposure time 20 s (39).

### Lentiviral Infection.

All of the transgenes were expressed in primary human keratinocytes by lentiviral transduction. Replication-defective and self-inactivating lentiviral vector (pCSII vector for EKAREV-nls and EKAREV-nes, pLenti vector for Involucrin-mCherry, and pCW57 vector for doxycyclin-inducible DUSP expression) were cotransfected with packaging plasmid (pCAG-HIVgp) and VSV-G/Rev−expressing plasmid (pCMV-VSVG-RSV-Rev) into HEK293T cells (Clontech). Cells expressing Involucrin-mCherry were selected by 2 µg/mL Puromycin treatment.

### siRNA Transfection.

For knockdown of β1 integrin, DUSP6, and DUSP10, SMART pool ON-TARGET plus siRNAs (Ambion/GE Healthcare) were used. The siRNAs were a mix of four sets of RNAi oligos.

For siRNA-mediated gene silencing, keratinocytes were cultured in KSFM containing 30 µg/mL BPE and 0.2 ng/mL EGF (Gibco) for 2 d. Keratinocytes were transfected using INTERFERin (Polyplus transfections) with the final siRNA concentration of 30 nM and 4 µL of INTERFERin reagent.

### In Situ Hybridization.

RNAscope Fluorescent Multiplex Assay (Advanced Cells Diagnostics; ACD) was used for in situ hybridization of human MAPK1, MAPK3, DUSP6, and DUSP10. NHKs were cultured in 24-well plates with feeders to allow colony formation. Following removal of the feeder layer, NHKs were fixed in 4% paraformaldehyde and subjected to protocols provided by ACD. For multiplex detection, samples were hybridized with probes against MAPK1and MAPK3 (C1) together with probes against DUSP6 or DUSP10 (C2).

### Microscopic Detection of In Situ Hybridization Signals.

Images were acquired on a Nikon A1R laser scanning confocal microscope with GaAsp detectors using a 40× CFI Plan Apo Lambda 0.95 NA objective (Nikon) and NIS-Elements (Nikon). Nuclear signal was excited by a 405-nm laser and detected by a 450/50-nm emission filter. MAPK1 and MAPK3 signals were excited by a 638-nm laser and detected by a 655/25-nm emission filter. DUSP6 or DUSP10 signal was excited by a 560-nm laser and detected by a 595/50-nm emission filter.

### Doxycycline-Inducible Overexpression.

Doxycycline-inducible gene expression constructs (pCW57-DUSP6, pCW57-DUSP10, and pCW57-MEK^EE^) were lentivirally cotransduced into NHKs with EKAREV or Involucrin-mCherry; 1 µg/mL doxycycline was added to the medium to induce DUSP expression. Live imaging was performed from 6 h after doxycycline treatment.

### Live Imaging of Human Keratinocytes.

The 2D culture images were acquired on a Nikon A1R laser scanning confocal microscope with GaAsp detectors using a 20× Plan Apo VC 0.75 NA objective (Nikon) and NIS-Elements (Nikon). Live cells were imaged in a temperature-controlled chamber (37 °C) in 5% CO_2_. For nuclear staining, Hoechst 33342 was added to the culture medium 30 min prior to imaging at a final concentration of 5 µg/mL. Images were acquired every 5 min for up to 24 h. For FRET imaging, the EKAREV biosensor was excited by a 445-nm laser, and 482/35-nm and 525/50-nm emission filters were used to acquire CFP and FRET images, respectively. Involucrin-mCherry was excited by a 560-nm laser and detected by a 595/50-nm emission filter. The Hoechst 33342 signal was excited by a 405-nm laser and detected by a 450/50-nm emission filter.

Images for cells on PDMS substrates were acquired on a Nikon A1R confocal/multiphoton laser scanning microscope with a 25× Apo LWD 1.1 NA objective. Mineral oil was used to cover the surface of the medium and prevent evaporation. Live cells were imaged in a temperature-controlled chamber (37 °C), and pH was maintained with 15 mM Hepes buffer. Images were acquired every 15 min.

### Single-Cell Proliferation Assay.

Mitotically inactivated J2-3T3 feeder cells were plated on collagen-coated 384-well glass bottom plates in complete FAD medium at the density of 20,000 cells per square centimeter. Single NHKs were plated onto each well. Six hours after plating wells that accommodated a single cell were subjected to live imaging to measure ERK pulse levels for 6 h. Cells were incubated for another 48 h, and the same wells were revisited by microscope to observe cell numbers.

### In Vivo Imaging of Mouse Epidermis.

Methods for live imaging of mouse epidermis were previously described (11). Briefly, the ear skin of anesthetized mice was depilated and stabilized between a cover glass and thermal conductive silicon gum sheet. In vivo imaging of tail epidermis was performed similarly. Depilated tail was flanked with two silicon gum sheets, and a cover glass was placed on the top. In vivo live imaging was performed with a ZEISS 7MP multiphoton microscope, equipped with a W Plan-Apochromat 20×/1.0 DIC VIS-IR M27 75-mm water immersion objective lens and a Coherent Chameleon Ti:Sapphire laser. EKAR-EVnls signal was detected by band pass filter (BP) 500 to 550 and BP 575 to 610 for CFP and FRET, respectively.

### FRET Analysis.

Single cell ERK activity was measured by ratiometry of CFP and FRET signals (FRET/CFP) since EKAREV functions by intramolecular FRET and the molecular number of the two fluorescence proteins is considered to be equal. Each signal level was measured by mean pixel intensity in individual cell areas.

### Automated Cell Tracking.

Tracking was performed by script-based operation of a FIJI plugin, TrackMate (https://imagej.net/TrackMate). FRET channel images were used for object detection and linking with manually optimized parameters. Identified object regions were redirected to corresponding CFP, FRET, and mCherry channel images to obtain mean intensities for each region. The whole dataset of XY location, time, and mean intensities was exported to Excel software (Microsoft Corporation) or MATLAB 2018Ra software (Mathworks) for further numerical analyses and data visualization.

### Quantification of In Situ Hybridization Signal.

The particle signals acquired by in situ hybridization were segmented by TrackMate. Individual cells were segmented by Watershed segmentation of NHK colony area based on nuclear positions. The whole dataset of XY location, mean intensity of in situ hybridization signal, and XY location of individual cells was exported to Excel software (Microsoft Corporation) or MATLAB 2018Ra software (Mathworks) for further numerical analyses and data visualization.

### Semiautomated Single-Cell Tracking of Cells Expressing EKAR-EVnes.

Cells expressing EKAR-EVnes cultured on the patterned PDMS substrate (Fig. 5*D*) were tracked in a semiautomated manner with a custom-made program for FIJI/ImageJ. Cellular center locations were tracked by eye based on the lack of nuclear signal of EKAR-EVnes. The 12-pixel square regions were automatically created around each center, and Huang’s fuzzy thresholding was applied to obtain cytoplasmic regions expressing EKAR-EVnes. The mean CFP and FRET signals were obtained for each region and used for ERK activity (FRET/CFP).

### Segmentation of Tip and Base Areas on the Patterned PDMS Substrate.

Tip and base regions were demarcated by circles with a diameter of 200 μm centered at each tip (Fig. 5*D*, white dotted circles). ERK^high^ and ERK^low^ cells in base areas (troughs) were gated by the 1.2 value of FRET/CFP (Fig. 5 *E*–*G*).

### Instantaneous Variance as a Measure of Population ERK Pulse Levels.

To quantify the level of ERK activity pulses of a population of cells at a given time point, we measured the variance of ERK activity (FRET/CFP) at that time point among all cells (instantaneous variance). An increase in the instantaneous variance indicates a higher variability of ERK activity in the population at a specific time point. Pulses were observed to behave as stochastic events, as suggested by the exponential distribution of interpulse intervals (*SI Appendix*, Fig. S1*D*). This, combined with the fact that the mean ERK activity did not change between conditions (Fig. 4*A*, Control/DUSP6) justifies the use of the instantaneous variance as a measure of the level of ERK activity pulses of a population at a specific time point. When the instantaneous variance remains unchanged between conditions (Fig. 4*B*, Control/DUSP10), we say that the level of ERK activity pulses is the same for both populations, regardless of changes in the mean level.

### Moving Variance as a Measure of ERK Pulse Levels in a Time Window.

In order to study the change in ERK activity pulses over time in individual cells, we analyzed overlapping moving time windows of 50 min. Each time window was small enough for the mean ERK activity, within the window, to be considered fixed, but long enough to accommodate an ERK activity pulse (typical pulse of ∼0.25 h). For each window, we computed the variance of ERK activity. The variance is a measure of dispersion of the measurements in the window, and quantifies the extent of the deviations of the signal from its mean value. These deviations can occur due to pulses, or noise (which is of much smaller amplitude than pulses). The variance captures both the amplitude and number of pulses in the time window, giving a quantitative measure of the pulsing level of ERK activity in the time window. The minimum value for the variance is zero, which corresponds to a signal without pulses or fluctuations; larger values of the variance indicate a higher level of pulsation.

This method allows a continuous assessment of the pulse levels over time. Other methods, such as peak detection and pulse count, amount to a discrete measurement of pulses, which does not lend itself naturally to a detailed quantitative analysis of the temporal evolution of ERK activity pulses.

### Phase Diagram.

To construct the phase diagram of ERK activity variance and Involucrin mean level, cells coexpressing EKAREV-nls, and the Involucrin reporter, Involucrin-mCherry, were considered. Time series obtained from automated cell tracking were analyzed by computing the variance of the ERK activity and the mean level of Involucrin on a moving window of 50 min. Only time series of more than 90 min were analyzed.

The values of variance of ERK activity and mean Involucrin level were plotted for every time window, providing a trajectory in the plane spanned by ERK activity variance and mean Involucrin level. This was repeated for all cells.

The ERK activity variance vs. Involucrin mean level plane was then divided into regular blocks. The trajectories that lay within each block were averaged to obtain a mean direction for each block. This procedure resulted in the phase diagram of ERK activity variance vs. mean Involucrin level. Every block in the phase diagram corresponds to a pair of ERK activity variance/Involucrin mean level values, while the arrow in the block indicates the mean direction to which these values changed in time.

The same procedure can be followed to construct other phase diagrams, such as mean ERK activity vs. mean Involucrin level.

### Phase Diagram Normalization and Transition Probabilities.

Each arrow in the phase diagram was decomposed into its *x* and *y* components. The *x* components were rescaled by the maximum value of the Involucrin mean level, that is, *x*′ = *x*/max(Inv. Mean level), while the *y* components were rescaled by the maximum value of the ERK activity variance, that is, *y*′ = *y*/max(ERK activity variance). This rescaling amounted to normalizing both axes of the phase diagram to the range [0,1], and allowed the comparison between the *x*′ and *y*′ components of the arrows.

The transition probabilities between neighboring blocks corresponded to *r*_*x*_ = *x*′/(|*x*′|+|*y*′|) and *r*_*y*_ = *y*′/(|*x*′|+|*y*′|), where |.| indicates the absolute value. Here, |*r*_*x*_| accounts for the probability of transitioning to the neighboring blocks of Involucrin mean level, while |*r*_*y*_| corresponds to the probability of transitioning to the neighboring blocks of ERK activity variance. The sign of *r*_*x,y*_ indicates the direction of the transition; a minus (plus) sign signifies a transition toward decreasing (increasing) values of Involucrin/ERK activity variance. The probabilities are normalized, such that |*r*_*x*_| + |*r*_*y*_| = 1.

### Cluster Analysis.

To analyze the spatial organization of cells in ear and tail epidermis, we measured the radial distribution function (RDF) g(*r*), which measures the deviations of the density of cells from that of a random distribution, as a function of the distance *r* from a cell of reference (62). To construct the RDF, we constructed rings of radius *r* and width Δ*r* around every cell of interest, and counted the total number N(*r*, Δ*r*) of cells that lie within the rings. We then constructed the reference number N_ref_(*r*, Δ*r*), which was computed by measuring N(*r*, Δ*r*) for a set of randomly distributed points in the field of interest. The field of interest was constructed by considering only regions of space where cells were observed in the experiment, as indicated by the black outline in Fig. 6 *G* and *M*, and *SI Appendix*, Fig. S6 *C* and *D*. This was done to prevent artifacts due to boundary effects that might bias the clustering results. Finally, we constructed the RDF as the ratio g(*r*) = N(r, Δ*r*)/N_ref_(*r*, Δ*r*).

We constructed the null RDF g_null_(*r*) = N_null_(*r*, Δ*r*)/N_ref_(*r*, Δ*r*), against which the experimental measurements were to be compared. In this case, both N_null_(*r*, Δ*r*) and N_ref_(*r*, Δ*r*) were measured for random distributions of points. In the null model, all particles are independent, randomly distributed points; hence the g_null_(*r*) is equal to unity. From 50 realizations of g_null_(*r*), we computed the 95% CIs. When the experimental RDF lies above (below) the 95% CI, the cells are considered clustered (dispersed) for that particular distance *r*, around cells.

The RDF was also used to study the clustering between two distinct populations, high and low pulsing cells. For this, we considered the cells of one group (high pulsing) as the reference cells around which the rings are constructed, while we counted the number of cells of the second group (low pulsing) that lie within the rings. In this case, when the experimental observations lie below (above) the 95% CI, both cell populations are considered segregated (grouped).

### Statistics.

Statistical analyses were performed using MS Excel or MATLAB R2018a (Mathworks) software. We made use of the two-tailed Student’s *t* test for unpaired data to quantify differences between experimental groups. Kolmogorov−Smirnov test was used to compare distributions. *P* values are indicated by * 0.01 < *P* < 0.05, ** 0.01 < *P* < 0.001, ***P < 0.001; n.s. = not significant.

## Supplementary Material

Supplementary File

Supplementary File

Supplementary File

Supplementary File

## Data Availability

All relevant data are available within the article text, *SI Appendix*, and Movies S1–S3.
