# Supplementary material for: Regulation of ERK basal and pulsatile activity control proliferation and exit from the stem cell compartment in mammalian epidermis
Source: Proc Natl Acad Sci U S A. 2020 Jul 10;117(30):17796–807. doi: 10.1073/pnas.2006965117 (PMC7395546; doi:10.1073/pnas.2006965117)
Supplement: Supplementary File [file pnas.2006965117.sapp.pdf]

Supporting Appendix

Fig. S1. Quantitative analysis of ERK activation pulses. (*A*) Schematic of the ERK pulse detection and quantification. Pulses are detected as local peaks with prominence larger than 0.05 FRET/CFP value. Pulse duration was determined as the width of pulse at half the prominence of each pulse. Interpulse interval was characterised as the latency period between pulses. (*B*) Validation of the quantification methods with kinase-dead EKAR-EVnls biosensor (EKAREV-TA-nls), where FRET does not occur. Left, proportion of pulsatile cells in keratinocytes expressing normal EKAR-EVnls or EKAREV-TA-nls. Right, histogram of ERK pulse frequencies in pulsatile cells detected in keratinocytes expressing EKAREV-TA-nls. Data were obtained from human keratinocytes, cultured on feeder layers in complete FAD medium. (*C*) Histogram of pulse durations, indicating the mean pulse width. (*D*) Histogram of interpulse intervals fitted to an exponential decay curve, showing the value of the decay rate τ. (*E* and *F*) Histogram of ERK pulse frequency (*E*) and basal ERK activity (*F*) in keratinocytes on Day3, Day5 and Day8. Asterisks indicate the two peaks in the histogram of ERK activity on Day3 (red) (n = 3,323 cells for Day3, 11,527 cells for Day5, 37,320 cells for Day8 cells). (*G*) Representative time-series of ERK activity of NHKs on Day2. (*H*) Heat-map of ERK activity over time for 50 cells ordered by descending mean ERK activity (FRET/CFP) over time. Colours indicate ERK activity. (*I* and *J*) Histogram of ERK pulse frequency (*I*) and basal ERK activity (*J*) in keratinocytes on Day2 compared to Day3 NHKs. (n = 542 cells for Day2, 3,323 cells for Day3). (*K*) Dot plots and correlation analysis of basal ERK activity and ERK pulse frequency in NHKs on the indicated days. Lines indicate regression lines and values are Spearman's rank correlation coefficient. (n = 542 cells for Day2, 3,323 cells for Day3, 11,527 cells for Day5, 37,320 cells for Day8 cells).

Fig. S2. ERK activity profile in stably high and low Involucrin expression. (*A* and *B*) Representative time-series of ERK activity (black) and Involucrin-mCherry expression (red) of cells expressing stably high (*A*), or low (*B*) Involucrin. Images of the cells of ERK activity and Involucrin are shown for the time points indicated by the orange circles in each time-series. Images are shown by the indicated LUTs below. (*C*) Dot plot of mean ERK activity and Involucrin-mCherry expression in HNK cells on different days (n = 3,323 cells for Day3, 11,527 cells for Day5, 37,320 cells for Day8 cells). (*D*) Phase diagram of ERK activity variance and mean activity obtained from n = 3,323 cells. Arrows indicate the direction of transition for each compartment.

Fig. S3. The effect of β1-integrin knockdown and Ca^{2+} chelation on ERK pulse patterns and differentiation. (*A* and *B*) Histograms of frequencies (*A*), and interpulse intervals (*B*), in cells treated with scrambled control siRNA (left) or β1 -integrin-targeted siRNA (right). Black dotted lines: mean. (n = 764 cells for control siRNA, 112 cells for β1 -integrin-targeted siRNA) (*C*) Frequency of ERK pulses. Data are shown by mean ± s.e.m (n = 764 cells for control siRNA, 112 cells for β1-integrin-targeted siRNA, Kolmogorov-Smirnov test; $P=7.9 \times 10^{-20}$). (*D*) Heat-map of ERK activity over time for 50 cells ordered by descending mean ERK activity (FRET/CFP) over time. Colours indicate ERK activity. (*E*) Representative time-series of ERK activity of cells treated with normal FAD medium (left) and Ca^{2+}-chelated medium (right). (*F* and *G*) Histograms of frequencies (*F*), and interpulse intervals (*G*) in cells cultured with Ca^{2+}-chelated medium. Black dotted lines: mean. (n = 757 cells for normal FAD treatment, 706 cells for Ca^{2+} chelated medium treatment). (*H*)

Frequency of ERK pulses. Data are shown by mean ± s.e.m (n = 757 cells for normal FAD treatment, 706 cells for Ca^{2+} chelated medium treatment, Kolmogorov-Smirnov test; P=0.0245). (*I*) Mean Involucrin reporter expression over time. NHK cells cultured for 5 days in complete FAD medium on feeders were given fresh FAD medium (black) or low Ca^{2+} FAD medium (red). Data are shown as mean ± s.e.m. Statistical significance was examined by Kolmogorov-Smirnov test; P values are indicated by ** P<0.01, *** P<0.001. (*J*) Heat-map of ERK activity over time for 50 cells ordered by descending mean ERK activity (FRET/CFP) over time. Colours indicate ERK activity. Cells were plated in KSFM medium (feeder-free, low Ca^{2+}) at low (2×10^4 /cm^2) or high (2×10^5 /cm^2) density and time-lapse images were acquired on Day1 after plating. (*K*) Representative time-series of ERK activity in cells cultured at low or high density as described in (*J*). (*L*) Frequency of ERK pulses. Data are shown by mean ± s.e.m (n = 51 cells for low cell density, 215 cells for high cell density, Kolmogorov-Smirnov test; P=2.3×10^{-10}).

Fig. S4. The effect of MEK inhibitor on ERK pulse patterns on different stem cell stages. (*A* and *C*) Heat-map of ERK activity over time for 50 cells ordered by descending mean ERK activity (FRET/CFP) over time. Colours indicate ERK activity. (*B* and *D*), Representative time-series of ERK activity of cells treated with DMSO or 1 μM MEK inhibitor, PD0325901 on day3 or day5 after plating. (*E*) Dot plots and correlation analysis of basal ERK activity and ERK pulse frequency in DMSO-treated NHKs on day3 (left) and day5 (right) after plating. Lines indicate regression lines and values are Spearman's rank correlation coefficient. (n = 644 cells for Day3 cells, 675 cells for Day5 cells). (*F*) Histogram of ERK activity in NHKs treated with DMSO on day3 (left) and day5 (right) after plating. (n = 644 cells for Day3 cells, 675 cells for Day5 cells). (*G*) Histogram of ERK frequency in NHKs treated with DMSO on day3 after plating (n = 644 cells). (*H*) Histogram of Involucrin-mCherry signal in each NHK cell treated with 1 μM MEKi on day3 after plating. The increase in Involucrin-mCherry signal was determined during live imaging for 13.6 hours. (n = 644 cells).

Fig. S5. The effect of DUSP6 overexpression on NHKs at different stages of differentiation. (*A* - *D*) Time-series examining cell populations binned by the initial Involucrin-mCherry fluorescence intensity: low (0 – 1000) (*A*), middle-low (1001 – 2000) (*B*), middle-high (2001 – 3000) (*C*), or high (3001 – 4095) (*D*). NHKs were treated with 1 μg/ml doxycyclin (green) to induce DUSP6 expression or with vehicle alone (black). The proportions of each population are normalised to the proportions at the start of recording.

Fig. S6. Clustering of cells with high or low ERK pulse frequency in mouse epidermis. (*A*) Heat-map of ERK activity over time for 50 cells ordered by descending mean ERK activity (FRET/CFP) over time in the basal (left) or suprabasal (right) layer of mouse epidermis. Colours indicate ERK activity. (*B*) Representative time-series of ERK activity in basal (left) or suprabasal (right) layer cells. (*C*) Clustering analysis of cells in the ear skin basal layer for the 700 most pulsatile (top) and 700 least pulsatile cells (middle) show the clustering between the most and least pulsatile subpopulations (bottom). The right panels show the radial distribution function g(r) (solid, blue line), null randomized (red lines), and the 95% c.i. (dotted lines). Values above or below the 95% c.i. indicate a significant clustering or dispersion of cells at the corresponding scale (distance), respectively. (*D*) The same analysis as (*C*), for the tail skin, where the 130 most and 130 least pulsatile cells were considered. The

black boundaries in the left panels of (*C*), and (*D*), enclose the area used to compute the null lines.

Movie S1. ERK activity pulses in cultured human keratinocytes. Human keratinocytes expressing EKAR-EVnls cultured on feeder cell layer for 5 days. Colours indicate ERK activity. Image size: 624 μm × 624 μm.

Movie S2. Time-lapse movie of human keratinocytes on the patterned PDMS substrate. Human keratinocytes expressing EKAR-EVnes cultured on the patterned PDMS substrate for 48 hours. Colours indicate ERK activity. Image size: 507 μm × 507 μm.

Movie S3. Time-lapse movie of the basal layer of mouse tail epidermis. Mouse tail epidermis expressing EKAR-EVnls FRET biosensor. Colours indicate ERK activity. Image size: 332 μm × 332 μm.

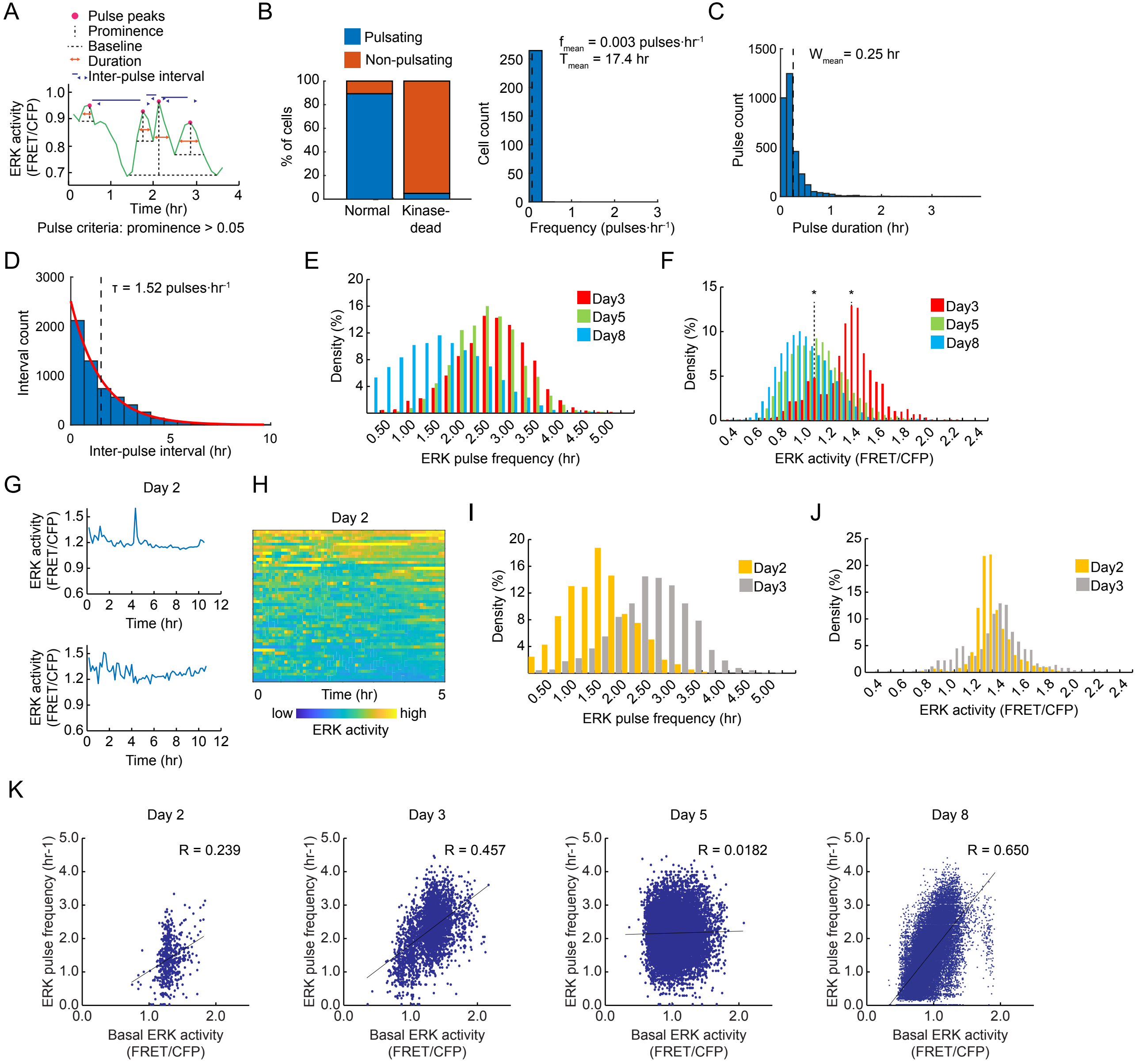

A
Pulse peaks
Prominence
Baseline
Duration
Inter-pulse interval
ERK activity (FRET/CFP)
Time (hr)
Pulse criteria: prominence > 0.05
B
Pulsating
Non-pulsating
% of cells
Normal
Kinase-dead
f_mean = 0.003 pulses·hr⁻¹
T_mean = 17.4 hr
Cell count
Frequency (pulses·hr⁻¹)
C
W_mean = 0.25 hr
Pulse count
Pulse duration (hr)
D
τ = 1.52 pulses·hr⁻¹
Interval count
Inter-pulse interval (hr)
E
Day3
Day5
Day8
Density (%)
ERK pulse frequency (hr)
F
Density (%)
ERK activity (FRET/CFP)
G
Day 2
ERK activity (FRET/CFP)
Time (hr)
H
Day 2
Time (hr)
low
high
ERK activity
I
Day2
Day3
Density (%)
ERK pulse frequency (hr)
J
Density (%)
ERK activity (FRET/CFP)
K
Day 2
R = 0.239
Day 3
R = 0.457
Day 5
R = 0.0182
Day 8
R = 0.650
ERK pulse frequency (hr-1)
Basal ERK activity (FRET/CFP)

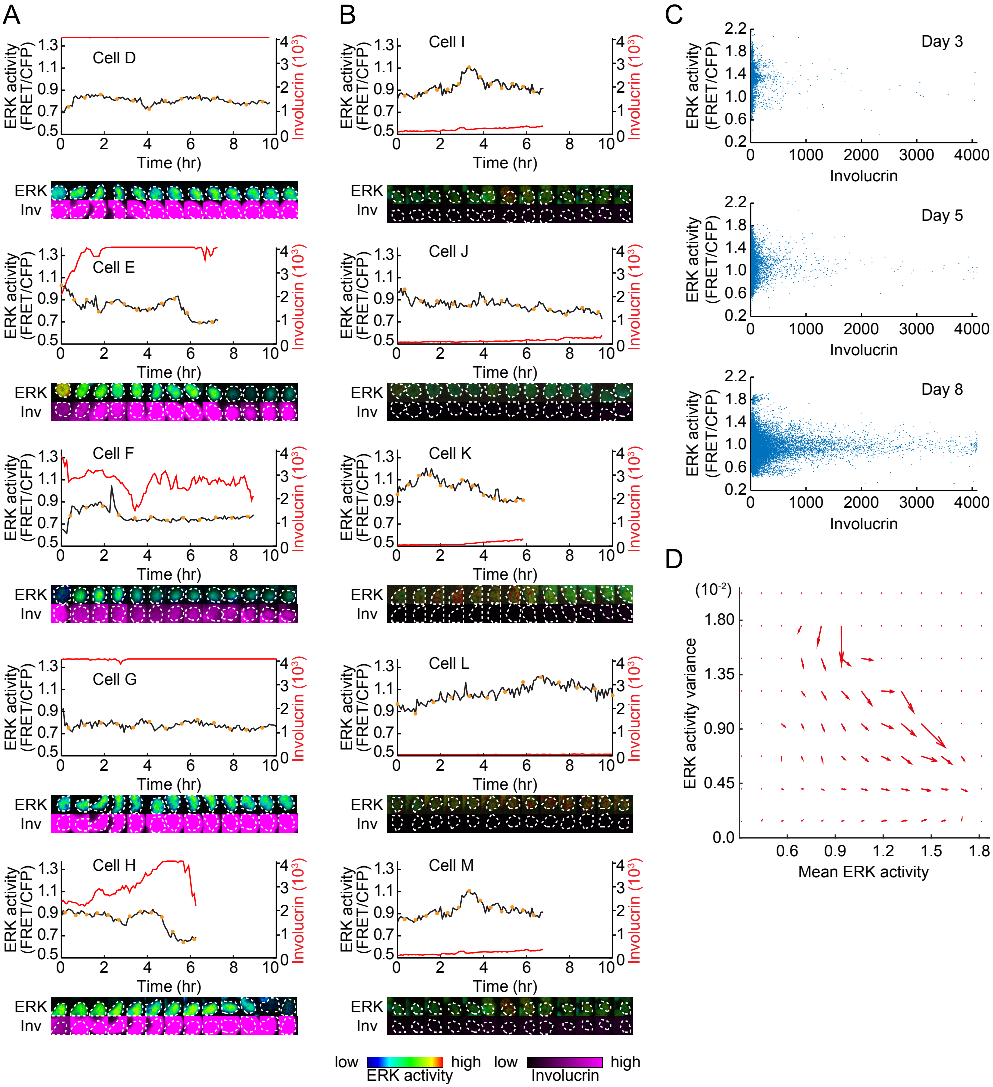

A
B
C
D
Cell D
Cell E
Cell F
Cell G
Cell H
Cell I
Cell J
Cell K
Cell L
Cell M
ERK activity (FRET/CFP)
Involucrin (10^3)
Time (hr)
ERK
Inv
Day 3
Day 5
Day 8
Involucrin
(10^{-2})
ERK activity variance
Mean ERK activity
low
high
ERK activity
low
high
Involucrin

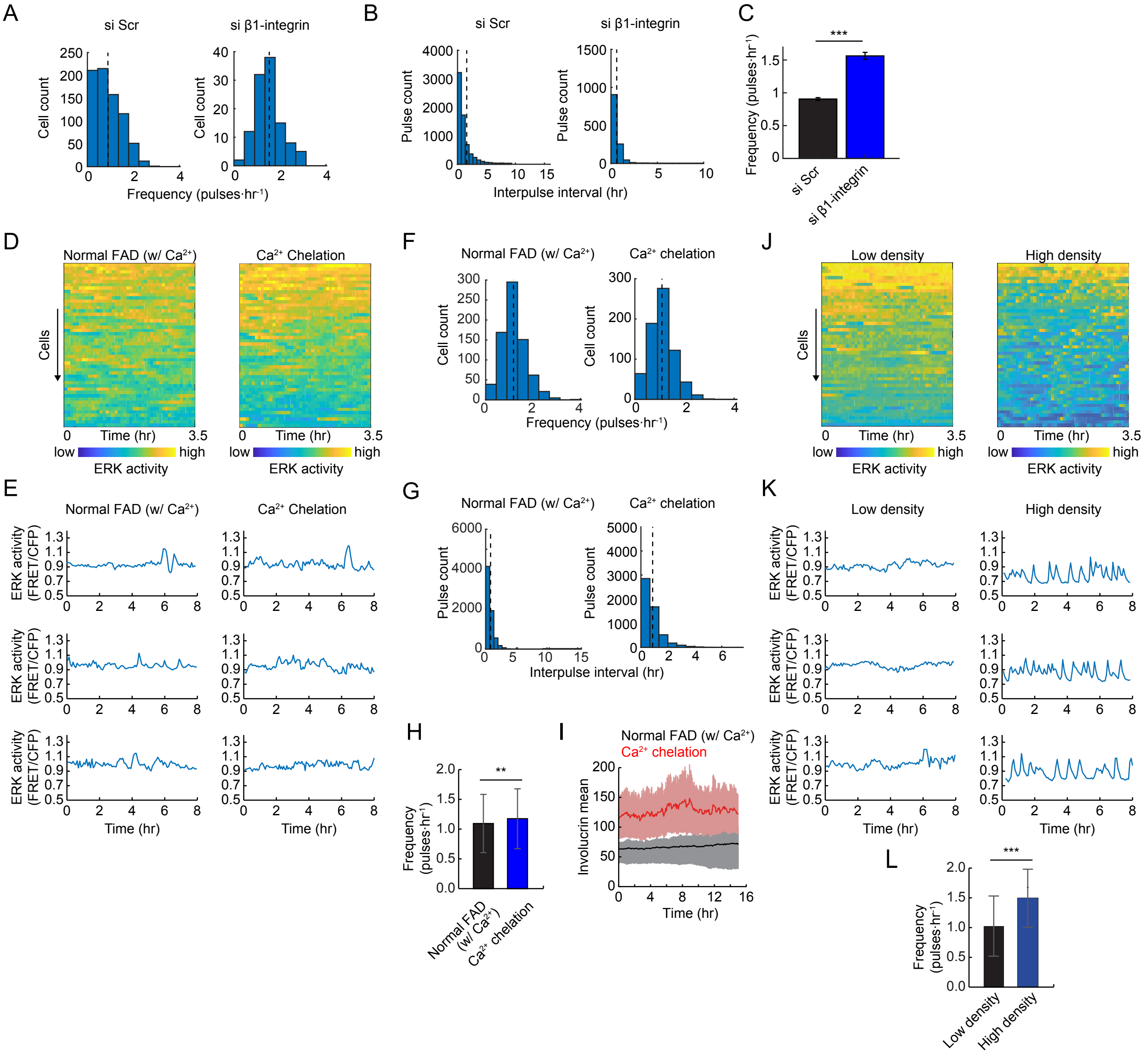
A
si Scr
si β1-integrin
Cell count
Frequency (pulses·hr-1)
B
Pulse count
Interpulse interval (hr)
C
***
Frequency (pulses·hr-1)
si Scr
si β1-integrin
D
Normal FAD (w/ Ca2+)
Ca2+ Chelation
Cells
Time (hr)
low
high
ERK activity
E
ERK activity (FRET/CFP)
Time (hr)
F
Normal FAD (w/ Ca2+)
Ca2+ chelation
Cell count
Frequency (pulses·hr-1)
G
Pulse count
Interpulse interval (hr)
H
**
Frequency (pulses·hr-1)
Normal FAD (w/ Ca2+)
Ca2+ chelation
I
Normal FAD (w/ Ca2+)
Ca2+ chelation
Involucrin mean
Time (hr)
J
Low density
High density
Cells
Time (hr)
ERK activity
K
Low density
High density
ERK activity (FRET/CFP)
Time (hr)
L
***
Frequency (pulses·hr-1)
Low density
High density

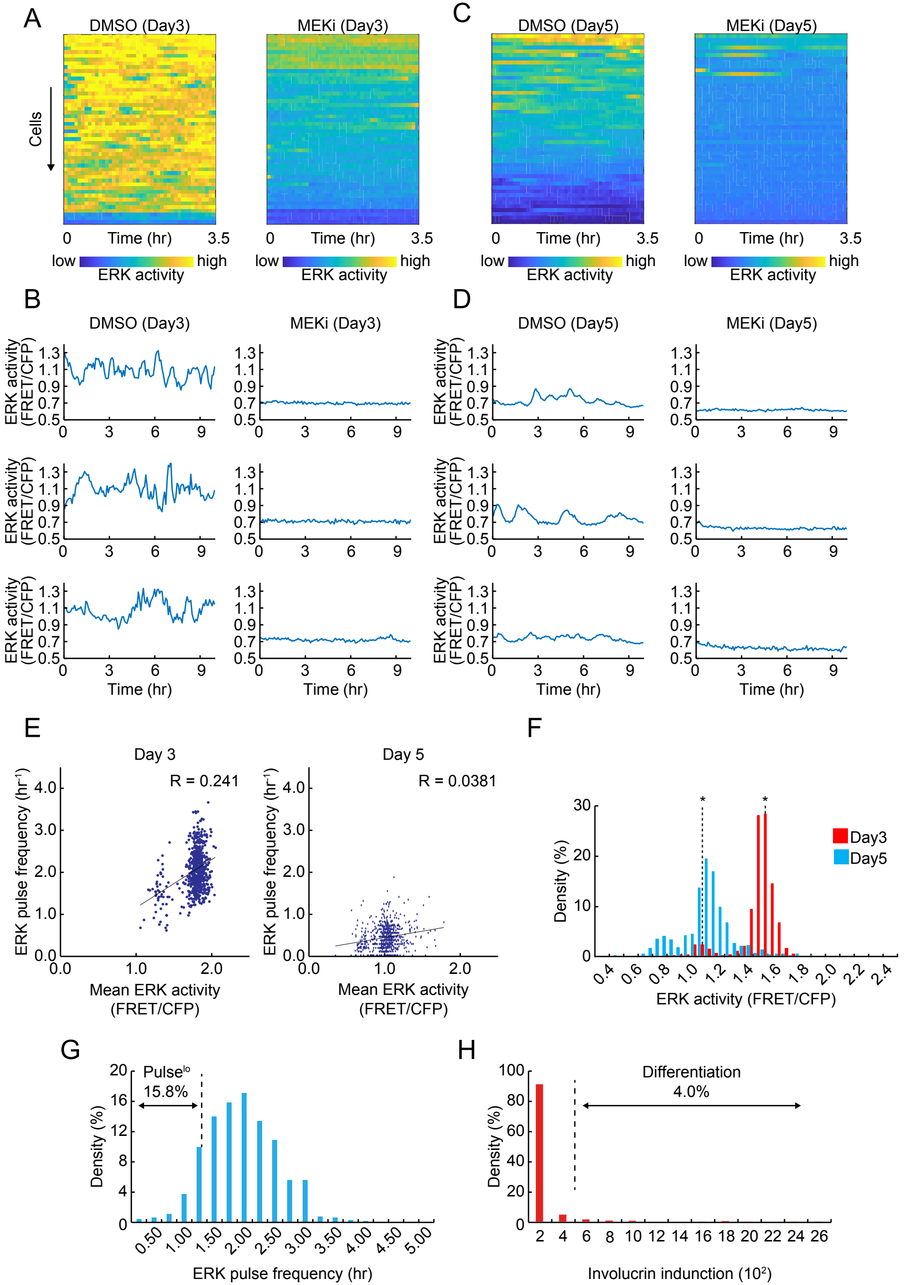
A
DMSO (Day3)
MEKi (Day3)
Cells
0
Time (hr)
3.5
low
high
ERK activity
C
DMSO (Day5)
MEKi (Day5)
B
D
ERK activity (FRET/CFP)
Time (hr)
E
Day 3
Day 5
R = 0.241
R = 0.0381
ERK pulse frequency (hr-1)
Mean ERK activity (FRET/CFP)
F
Day3
Day5
Density (%)
ERK activity (FRET/CFP)
G
Pulselo 15.8%
Density (%)
ERK pulse frequency (hr)
H
Differentiation 4.0%
Density (%)
Involucrin indunction (102)

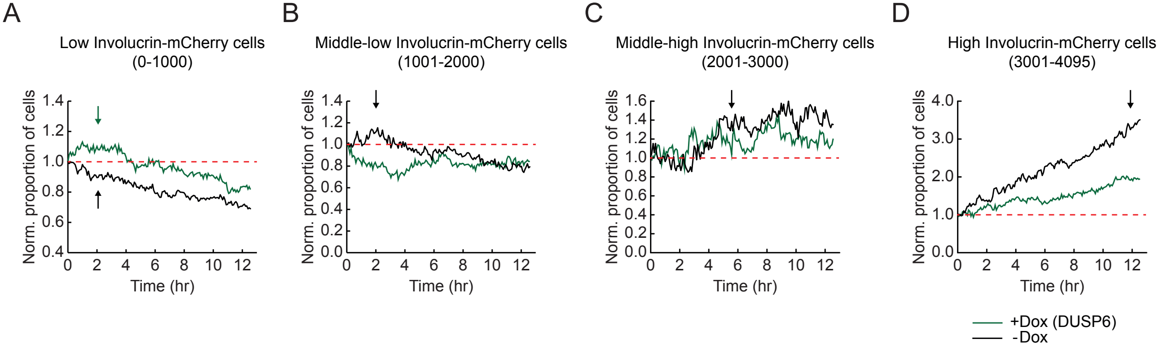

A
Low Involucrin-mCherry cells
(0-1000)
Norm. proportion of cells
1.4
1.2
1.0
0.8
0.6
0.4
0
2
4
6
8
10
12
Time (hr)
B
Middle-low Involucrin-mCherry cells
(1001-2000)
Norm. proportion of cells
1.4
1.2
1.0
0.8
0.6
0.4
0.2
0.0
0
2
4
6
8
10
12
Time (hr)
C
Middle-high Involucrin-mCherry cells
(2001-3000)
Norm. proportion of cells
1.6
1.4
1.2
1.0
0.8
0.6
0.4
0.2
0.0
0
2
4
6
8
10
12
Time (hr)
D
High Involucrin-mCherry cells
(3001-4095)
Norm. proportion of cells
4.0
3.0
2.0
1.0
0.0
0
2
4
6
8
10
12
Time (hr)
+Dox (DUSP6)
-Dox

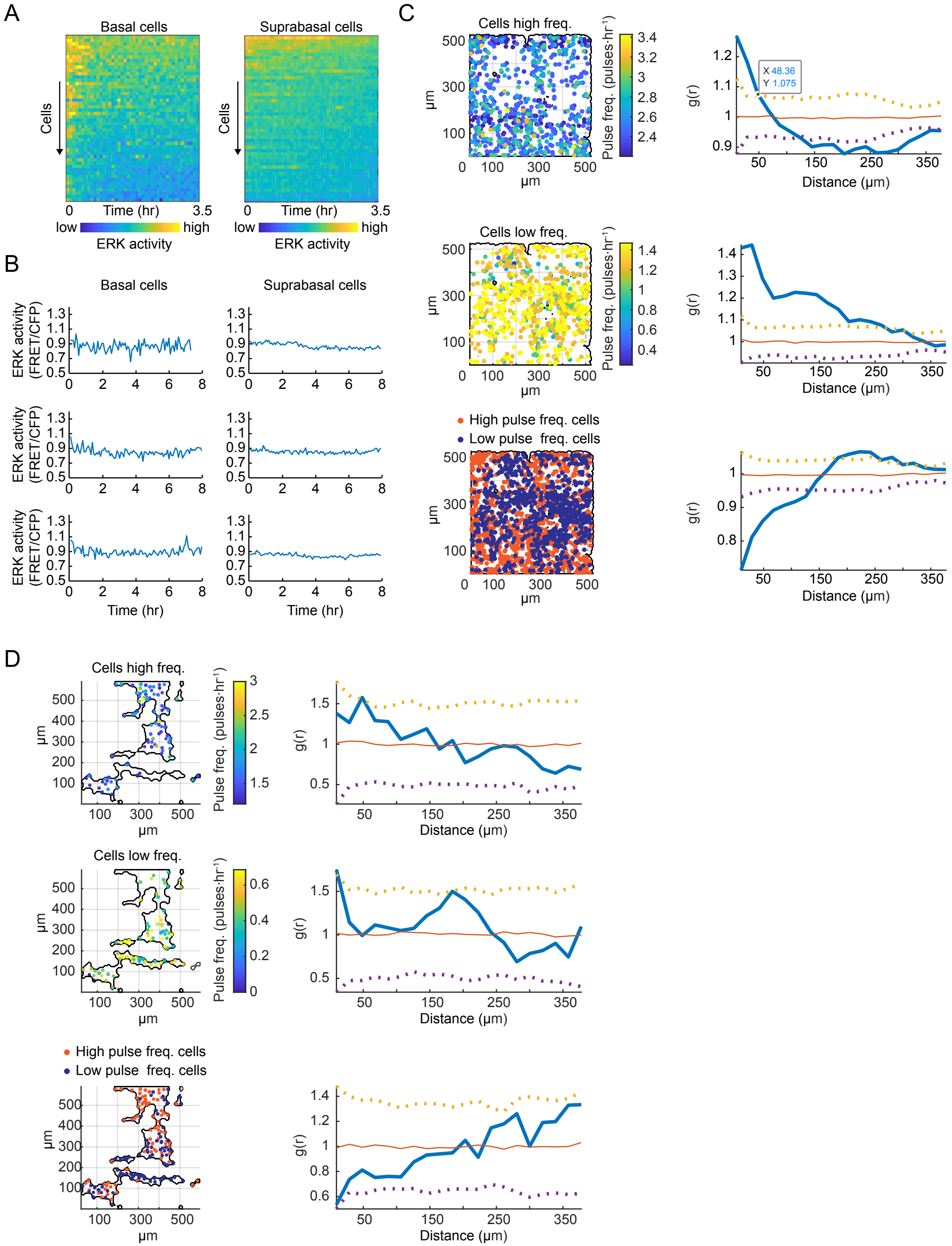

A
Basal cells
Suprabasal cells
Cells
Time (hr)
0
3.5
low
high
ERK activity
B
Basal cells
Suprabasal cells
ERK activity (FRET/CFP)
1.3
1.1
0.9
0.7
0.5
0
2
4
6
8
Time (hr)
C
Cells high freq.
Pulse freq. (pulses·hr-1)
3.4
3.2
3
2.8
2.6
2.4
500
300
100
µm
g(r)
X 48.36
Y 1.075
Distance (µm)
Cells low freq.
1.4
1.2
1
0.8
0.6
0.4
High pulse freq. cells
Low pulse freq. cells
D
Cells high freq.
Pulse freq. (pulses·hr-1)
3
2.5
2
1.5
Cells low freq.
0.6
0.4
0.2
0
High pulse freq. cells
Low pulse freq. cells
